# Supplementary material for: Integrating chemical structures as treatments improves representations of microscopy images for morphological profiling
Source: PLoS Comput Biol. 2026 Jul 29;22(7):e1014483. doi: 10.1371/journal.pcbi.1014483 (PMC13446733; doi:10.1371/journal.pcbi.1014483)
Supplement: Supplementary file 1 [file pcbi.1014483.s001.pdf]

S1 Text: Supplementary Methods

Permutation Tests for Mechanism of Action Enrichment

To quantify retrieval performance, we measure whether the top-ranked matches for a query are significantly enriched for the same mechanism of action compared to non-hits. Specifically, for a retrieval set defined by a cut-off k, let a be the number of matching class samples retrieved, b be the number of non-matching samples retrieved, c be the number of matching samples not retrieved, and d be the number of non-matching samples not retrieved. The Odds Ratio (OR) is conventionally defined as:

$$OR = \frac{a \cdot d}{b \cdot c} \tag{1}$$

Besides reporting the raw OR, we also report the percentage of classes that achieve statistically significant enrichment. Significance is determined by comparing the observed Odds Ratio (OR_{obs}) against the Odds Ratios of randomly selected samples (OR_{rand}) of the same set size. Using $M = 100$ random permutations, the p-value for the j-th query is calculated as:

$$p_j = \frac{1}{M} \sum_{i=1}^{M} \mathcal{I}(OR_{rand}^{(i)} \geq OR_{obs}) \tag{2}$$

where $\mathcal{I}$ is the indicator function. Finally, we calculate the percentage of significant queries (P_{sig}) across the entire dataset. Let N be the total number of queries. The metric is defined as the fraction of queries where the permutation test yields a p-value below the significance threshold $\alpha = 0.05$:

$$P_{sig} = \frac{\sum_{j=1}^{N} \mathcal{I}(p_j < 0.05)}{N} \times 100 \tag{3}$$
