# Supplementary material for: Integrating chemical structures as treatments improves representations of microscopy images for morphological profiling
Source: PLoS Comput Biol. 2026 Jul 29;22(7):e1014483. doi: 10.1371/journal.pcbi.1014483 (PMC13446733; doi:10.1371/journal.pcbi.1014483)
Supplement: Supplementary file 2 [file pcbi.1014483.s002.pdf]

Table A. Top-1 Not-Same-Batch (NSB) / Not-Same-Source (NSS) 1-NN Retrieval Accuracy across the 4 benchmarking settings for the POS-CTL test datasets, identical to the results in Figure 4 and reproduced in numerical form for reproducibility purposes. Error bars represent the standard deviation across n=3 replicates with random seeds. Asterisk indicates that MICON significantly outperforms the baseline, defined as $p < 0.05$ on an unpaired one-tailed t-test with performance across the n=3 random seeds as trials.

POS-CTL(ID)	1-NN NSB	1-NN NSS	1-NN NSB (Unseen Source)	1-NN NSS (Unseen Source)
Random Chance			12.50	
CellProfiler	$36.56 \pm 1.16^*$	$9.32 \pm 0.79^*$	$39.26 \pm 1.17^*$	$7.42 \pm 0.79^*$
ResNet(Transfer)	$34.73 \pm 1.33^*$	$7.41 \pm 0.63^*$	$32.95 \pm 1.07^*$	$7.31 \pm 0.71^*$
DINOv3(Transfer)	$35.84 \pm 1.18^*$	$8.47 \pm 0.62^*$	$36.41 \pm 1.21^*$	$7.36 \pm 0.74^*$
OpenPhenom-S/16	$35.41 \pm 1.07^*$	$8.22 \pm 0.51^*$	$38.77 \pm 1.12^*$	$7.42 \pm 0.63^*$
SimCLR	$37.77 \pm 1.18^*$	$16.56 \pm 0.95^*$	$34.45 \pm 1.32^*$	9.12 ± 0.86
CLIP	$34.33 \pm 1.46^*$	$12.16 \pm 0.88^*$	$32.53 \pm 1.34^*$	10.76 ± 1.04
DINOv3	$42.41 \pm 0.97^*$	$19.21 \pm 0.78^*$	$33.54 \pm 1.17^*$	10.97 ± 0.71
MICON (BAC Loss Only)	$56.57 \pm 1.65^*$	$25.21 \pm 1.16^*$	$48.13 \pm 1.41^*$	10.23 ± 1.26
MICON	$\mathbf{62.24 \pm 1.41}$	$\mathbf{28.43 \pm 1.05}$	$\mathbf{51.57 \pm 1.66}$	$\mathbf{11.25 \pm 1.18}$

Table B. Top-1 Not-Same-Batch (NSB) / Not-Same-Source (NSS) 1-NN retrieval accuracy across the 4 benchmarking settings for the TGT2 test datasets, identical to the results in 4 and reproduced in numerical form for reproducibility purposes. Error bars represent the standard deviation across n=3 replicates with random seeds. Asterisk indicates that MICON significantly outperforms the baseline, defined as $p < 0.05$ on an unpaired one-tailed t-test with performance across the n=3 random seeds as trials.

TGT-2 (ID)	1-NN NSB (ID)	1-NN NSS (ID)	1-NN NSB (Unseen Compound)	1-NN NSS (Unseen Compound)
Random Chance	0.33		0.21	
CellProfiler	$5.03 \pm 0.43^*$	$0.67 \pm 0.17^*$	$3.53 \pm 0.29^*$	$1.66 \pm 0.29^*$
ResNet(Transfer)	$2.34 \pm 0.26^*$	$0.21 \pm 0.03^*$	$1.97 \pm 0.16^*$	$0.37 \pm 0.02^*$
DINOv3(Transfer)	$3.82 \pm 0.31^*$	$0.45 \pm 0.06^*$	$2.61 \pm 0.21^*$	$0.86 \pm 0.09^*$
OpenPhenom-S/16	$4.26 \pm 0.33^*$	$0.56 \pm 0.04^*$	$2.69 \pm 0.14^*$	$0.96 \pm 0.07^*$
SimCLR	$5.61 \pm 0.75^*$	$1.42 \pm 0.15^*$	$2.17 \pm 0.27^*$	$0.42 \pm 0.12^*$
CLIP	$6.22 \pm 0.64^*$	$2.99 \pm 0.36^*$	$2.33 \pm 0.26^*$	$0.99 \pm 0.16^*$
DINOv3	$8.45 \pm 0.57^*$	3.34 ± 0.22	$2.75 \pm 0.31^*$	1.37 ± 0.19
MICON (BAC Loss Only)	11.17 ± 0.89	3.65 ± 0.47	3.76 ± 0.43	1.79 ± 0.25
MICON	$\mathbf{11.89 \pm 1.04}$	$\mathbf{3.93 \pm 0.55}$	$\mathbf{4.92 \pm 0.65}$	$\mathbf{2.15 \pm 0.33}$

Table C. Top-1 Not-Same-Batch (NSB) / Not-Same-Source (NSS) 1-NN Retrieval Accuracy across the 4 benchmarking settings for the POS-CTL test datasets after batch correction post-processing, identical to the results in Figure 5and reproduced in numerical form for reproducibility purposes. Error bars represent the standard deviation across n=3 replicates with random seeds. Asterisk indicates that MICON significantly outperforms the baseline, defined as $p < 0.05$ on an unpaired one-tailed t-test with performance across the n=3 random seeds as trials.

POS-CTL(ID)	1-NN NSB	1-NN NSS	1-NN NSB (Unseen Source)	1-NN NSS (Unseen Source)
Random Chance			12.50	
CellProfiler	$55.29 \pm 0.63^*$	34.62 ± 0.52	53.34 ± 1.87	$30.41 \pm 0.52^*$
ResNet(Transfer)	$47.73 \pm 0.97^*$	$30.47 \pm 0.44^*$	$42.14 \pm 1.17^*$	$24.57 \pm 0.65^*$
DINOv3(Transfer)	$52.43 \pm 0.82^*$	$32.84 \pm 0.49^*$	$48.62 \pm 1.31^*$	$27.83 \pm 0.58^*$
OpenPhenom-S/16	$56.32 \pm 0.52^*$	$34.98 \pm 0.41^*$	$52.17 \pm 1.19^*$	$30.17 \pm 0.34^*$
SimCLR	$58.64 \pm 1.06^*$	$33.49 \pm 0.54^*$	$47.15 \pm 1.55^*$	$25.97 \pm 0.68^*$
CLIP	$56.46 \pm 1.22^*$	$32.17 \pm 0.68^*$	$43.15 \pm 1.63^*$	$23.46 \pm 0.78^*$
DINOv3	$60.17 \pm 1.45^*$	34.22 ± 0.62	$51.24 \pm 1.31^*$	$27.55 \pm 0.52^*$
MICON	$\mathbf{63.18 \pm 1.53}$	$\mathbf{35.36 \pm 0.51}$	$\mathbf{54.84 \pm 1.54}$	$\mathbf{32.52 \pm 0.61}$

Table D. Top-1 Not-Same-Batch (NSB) / Not-Same-Source (NSS) 1-NN retrieval accuracy across the 4 benchmarking settings for the TGT2 test datasets after batch correction post-processing, identical to the results shown in Figure 5 and reproduced in numerical form for reproducibility purposes. Error bars represent the standard deviation across n=3 replicates with random seeds. Asterisk indicates that MICON significantly outperforms the baseline, defined as $p < 0.05$ on an unpaired one-tailed t-test with performance across the n=3 random seeds as trials.

TGT-2 (ID)	1-NN NSB (ID)	1-NN NSS (ID)	1-NN NSB (Unseen Compound)	1-NN NSS (Unseen Compound)
Random Chance	0.33		0.21	
CellProfiler	$11.5 \pm 0.32^*$	$3.16 \pm 0.21^*$	$5.54 \pm 0.23^*$	$3.11 \pm 0.12^*$
ResNet(Transfer)	$8.17 \pm 0.15^*$	$2.26 \pm 0.14^*$	$4.33 \pm 0.17^*$	$2.15 \pm 0.11^*$
DINOv3(Transfer)	$9.86 \pm 0.21^*$	$2.75 \pm 0.16^*$	$4.94 \pm 0.19^*$	$2.67 \pm 0.14^*$
OpenPhenom-S/16	$11.85 \pm 0.17^*$	$3.65 \pm 0.18^*$	$5.47 \pm 0.13^*$	$3.16 \pm 0.12^*$
SimCLR	$9.61 \pm 0.53^*$	$3.58 \pm 0.15^*$	$5.32 \pm 0.27^*$	$2.13 \pm 0.23^*$
CLIP	$11.69 \pm 0.37^*$	$3.99 \pm 0.11^*$	$5.79 \pm 0.26^*$	$2.36 \pm 0.15^*$
DINOv3	13.19 ± 0.33	4.75 ± 0.09	5.97 ± 0.16	2.97 ± 0.12
MICON	$\mathbf{13.85 \pm 0.49}$	$\mathbf{5.17 \pm 0.18}$	$\mathbf{7.23 \pm 0.33}$	$\mathbf{3.98 \pm 0.15}$

Table E. Detailed metadata statistics for the POS-CTL data splits shown in Figure 1.

	Perturbations	# Sources	Source IDs	Batches	Plates	Wells	FOVs
ID Training Dataset	8	6	2,3,5,7,8,11	57	679	21284	395279
ID Validation Dataset	8	6	2,3,5,7,8,11	6	139	4305	89988
ID Test Dataset	8	6	2,3,5,7,8,11	12	283	9078	192094
Unseen Sources OOD Training Dataset	8	5	3,5,7,8,11	57	756	23752	479556
Unseen Sources OOD Validation Dataset	8	5	3,5,7,8,11	5	137	4254	92535
Unseen Sources OOD Query Dataset	8	1	2	8	133	4096	60853
Unseen Sources OOD Retrieval Dataset	8	1	2	5	86	2565	44417

Table F. Detailed metadata statistics for the Target-2 data splits as shown in Figure 2.

	Perturbations	# Sources	Source IDs	Batches	Plates	Wells	FOVs
ID Training Dataset	301	10	2,3,4,5,6,7,8,9,11,13	71	92	35156	325834
ID Validation Dataset	301	10	2,3,4,5,6,7,8,9,11,13	10	15	5723	53341
ID Test Dataset	301	10	2,3,4,5,6,7,8,9,11,13	20	28	10875	99781
Unseen Compounds OOD Training Dataset	301	10	2,3,4,5,6,7,8,9,11,13	91	122	46667	432500
Unseen Compounds OOD Validation Dataset	301	10	2,3,4,5,6,7,8,9,11,13	10	13	5087	46456
Unseen Compounds OOD Query Dataset	184	8	2,3,5,6,7,8,9,11	70	324	493	4053
Unseen Compounds OOD Retrieval Dataset	184	8	2,3,5,6,7,8,9,11	54	222	368	3079

Table G. Hyperparameters of the MICON architecture.

Model		Training	
ECFP4 fingerprint dimension	2048	optimizer	Adam
image encoder embedding size	1000	batch size	64
image projector hidden size	512	learning rate	1e-3
molecule hidden size	512	warmup steps	2000
fusion module hidden size	512	weight decay	1e-2
final projected embedding size	256	gradient clipping	1.0

Table H. Top-1/3/5 Not-Same-Batch (NSB) / Not-Same-Source (NSS) 1-NN Retrieval Accuracy across the 4 benchmarking settings for the POS-CTL test datasets

POS-CTL(ID) Top-1/3/5	1-NN NSB	1-NN NSS	1-NN NSB (Unseen Source)	1-NN NSS (Unseen Source)
Random Chance			12.50	
CellProfiler	36.6/38.2/40.7	9.3/10.2/10.9	39.3/41.2/43.5	7.4/7.9/8.2
ResNet(Transfer)	34.7/35.2/37.5	7.4/7.6/7.6	33.0/33.5/36.2	7.3/7.3/7.5
SimCLR	37.8/38.6/38.6	16.6/16.9/16.9	34.5/34.5/34.9	9.1/9.1/9.3
CLIP	34.3/34.7/35.5	12.2/13.1/13.9	32.5/33.6/33.9	10.8/11.2/11.2
DINOv3	42.4/43.6/44.9	19.2/19.5/20.2	33.5/33.9/34.3	11.0/11.8/12.5
MICON (BAC Loss Only)	56.6/58.2/58.9	25.2/26.3/26.6	48.1/49.2/49.7	10.2/10.2/10.6
MICON	**62.2/64.7/65.5**	**28.4/29.6/30.4**	**51.6/53.1/53.7**	**11.3/13.3/14.7**

Table I. Top-1/3/5 Not-Same-Batch (NSB) / Not-Same-Source (NSS) 1-NN retrieval accuracy across the 4 benchmarking settings for the TGT2 test datasets

TGT-2 (ID)	1-NN NSB (ID)	1-NN NSS (ID)	1-NN NSB (Unseen Compound)	1-NN NSS (Unseen Compound)
Random Chance	0.33		0.21	
CellProfiler	5.03/5.89/7.33	0.67/0.67/1.12	3.53/3.72/3.72	1.66/1.79/1.79
ResNet(Transfer)	2.34/2.34/2.79	0.21/0.37/0.76	1.97/1.97/1.97	0.37/0.37/0.37
SimCLR	5.61/5.61/6.38	1.42/1.42/1.42	2.17/2.17/2.35	0.42/0.42/0.42
CLIP	6.22/6.76/7.44	2.99/2.99/3.68	2.33/2.33/2.33	0.99/0.99/0.99
DINOv3	8.45/8.96/9.34	3.34/3.34/3.65	2.75/2.75/2.94	1.37/1.37/1.46
MICON (BAC Loss Only)	11.17/12.36/13.19	3.65/3.74/3.74	3.76/3.76/3.76	1.79/1.79/2.35
MICON	**11.89/12.97/14.37**	**3.93/4.35/4.35**	**4.92/5.82/5.82**	**2.15/2.15/2.35**

Table J. Top-1/3/5 Not-Same-Batch (NSB) / Not-Same-Source (NSS) 1-NN Retrieval Accuracy across the 4 benchmarking settings the POS-CTL test datasets after batch correction post-processing

POS-CTL(ID)	1-NN NSB	1-NN NSS	1-NN NSB (Unseen Source)	1-NN NSS (Unseen Source)
Random Chance			12.50	
CellProfiler	55.3/58.2/63.5	34.6/37.5/40.1	53.3/57.2/62.6	30.4/33.9/36.7
ResNet(Transfer)	47.7/49.5/50.2	30.4/32.6/34.1	42.1/45.5/48.6	24.6/27.3/31.6
SimCLR	58.64/60.6/64.2	33.5/35.9/37.1	47.2/50.4/55.4	26.0/28.5/29.3
CLIP	56.5/59.3/62.9	32.2/35.1/36.9	43.3/48.9/53.1	23.5/25.2/28.4
DINOv3	60.2/63.5/66.3	34.2/37.8/39.2	51.2/53.4/57.3	27.6/30.0/35.5
MICON	**63.2/65.1/68.5**	**35.4/38.5/39.4**	**54.9/55.9/58.2**	**32.5/33.2/35.9**

Table K. Top-1 Not-Same-Batch (NSB) / Not-Same-Source (NSS) 1-NN retrieval accuracy across the 4 benchmarking settings for the TGT2 test datasets after batch correction post-processing

TGT-2 (ID)	1-NN NSB (ID)	1-NN NSS (ID)	1-NN NSB (Unseen Compound)	1-NN NSS (Unseen Compound)
Random Chance	0.33		0.21	
CellProfiler	11.5/12.36/14.75	3.16/3.79/4.86	5.54/6.37/8.51	3.11/4.86/5.75
ResNet(Transfer)	8.17/10.32/10.86	2.26/2.82/3.16	4.33/4.76/5.54	2.15/2.15/3.73
SimCLR	9.61/10.56/12.73	3.58/3.97/4.64	5.32/5.54/5.79	2.13/3.11/3.73
CLIP	11.69/12.36/12.96	3.99/4.32/5.17	5.79/5.93/6.22	2.36/3.34/3.95
DINOv3	13.19/13.95/14.75	4.75/5.32/5.86	5.97/6.34/6.79	2.97/3.73/4.32
MICON	**13.85/14.75/15.98**	**5.17/5.86/6.65**	**7.23/7.52/8.16**	**3.98/4.86/5.96**
