# Supplementary material for: Integrating chemical structures as treatments improves representations of microscopy images for morphological profiling
Source: PLoS Comput Biol. 2026 Jul 29;22(7):e1014483. doi: 10.1371/journal.pcbi.1014483 (PMC13446733; doi:10.1371/journal.pcbi.1014483)
Supplement: Supplementary file 3 [file pcbi.1014483.s008.pdf]

Review Report

Title of the Manuscript: Causal integration of chemical structures improves representations of microscopy images for morphological profiling

Manuscript ID: PCOMPBIOL-D-25-00920

Summary and Significance

This manuscript fits within the scope of PLOS Computational Biology, presenting advancements in representation learning of cellular phenotypes resulting from chemical perturbations. Specifically, the authors propose a novel self-supervised framework that incorporates chemical structural data, thus improving the robustness and quality of learned representations. The approach is original and addresses a need for more discriminative and informative representation of morphological profiles.

Detailed Comments for Authors

Strengths:

- Original methodology: Effectively addresses significant challenges in contrastive learning involving cellular images and chemical structures.
- Innovative fusion module: Intelligently integrates chemical structural data into phenotypic profile representations, effectively utilizing numerous positive control images.
- Relevant loss function: The proposed loss is well-aligned with the stated learning objectives.
- Robust ablation study: Clearly demonstrates the individual contributions of each loss component, validating their effectiveness.
- Strategic evaluation design: Utilizes Out-of-Distribution (OOD) Unseen Sources Splits, which effectively assess the generalization capabilities and real-world utility of the model.
- Performance: Outperform all baselines in the proposed evaluation scenarios.
- Practical applicability: Demonstrates the model's capability (MICON) to synthesize phenotypic effects of previously unseen chemical compounds, showcasing practical utility.

- High readability: Well-organized manuscript with logical flow and clear explanations.
- Transparency and reproducibility: documented training details, including hyperparameters and computational hardware.
- Open access resources: Data and code are publicly available, facilitating reproducibility and further research.

Weaknesses and Recommendations:

- Need for stronger baseline with same training and evaluation data:
    - Transfer Learning (from natural images) is a strong and simple baseline.
    - The current baseline (SimCLR) may not fully reflect state-of-the-art performance in self-supervised learning for morphological profiling. DINOv2 has shown superior performance in recent literature (see https://www.nature.com/articles/s41598-025-88825-4) and should thus be included as a stronger and more relevant baseline.
- The evaluation could be extended to more task than replicate retrieval in order to assess the quality of the extracted biological signal :
    - Mechanism of Action classification (see https://www.nature.com/articles/s41467-024-45999-1)
    - Drug Target Interaction and known biological relationship recall (see: https://arxiv.org/pdf/2503.20158) highlighting the model's utility for practical biological understanding.
- While synthesizing phenotypic effects for unseen compounds is highly valuable, a comparison to a more conventional and widely recognized model like CLIP (contrastive language-image pre-training) would provide a clearer benchmark for assessing innovation and performance advantages.

Minor Points

- As discussed by the authors, drug screening experiments typically include many compounds with minimal phenotypic impacts relative to negative controls. It would thus be valuable to specifically evaluate the model on such low-impact compounds to enhance real-world applicability.
- Similarly, evaluating the model with varying numbers of images per compound—as suggested in the discussion—would strengthen the generalizability of results.
- Comparing methods that inherently correct batch effects (such as MICON) directly to methods without built-in batch correction (e.g., CellProfiler) without standard post-processing normalization may not provide a fair comparison.
- When batch-effect correction is applied, the chosen approach for CellProfiler features may not fully represent the current state-of-the-art methods (see https://www.nature.com/articles/s41467-024-50613-5).

- In the associated GitHub repository (github.com/microsoft/MICON), the provided link to "the official JUMP Cell Painting dataset" actually refers to the method proposed by Watkinson et al. (https://ieeexplore.ieee.org/document/10635200). This should be properly cited to avoid confusion.
- There are a few typo:
  - Line 58: "ifmages" → "images"
  - Line 106: "(cpg0016, see Section)"—section reference is missing or incomplete (likely "Methods").

Final Recommendation:

The manuscript presents strong contributions and significant innovation. However, minor revisions are required, specifically:

- **Incorporating additional evaluation tasks (e.g., Mechanism of Action classification, drug-target interaction recall) to better assess biological utility.**
- **Benchmarking against stronger (DINOv2 in SSL, CLIP for phenotype prediction) and simple (pre-trained DINOv2-G) baselines to substantiate claims of methodological improvement.**

Once these revisions are addressed, the manuscript will be suitable for publication.
