# Supplementary material for: Integrating chemical structures as treatments improves representations of microscopy images for morphological profiling
Source: PLoS Comput Biol. 2026 Jul 29;22(7):e1014483. doi: 10.1371/journal.pcbi.1014483 (PMC13446733; doi:10.1371/journal.pcbi.1014483)
Supplement: Supplementary file 4 [file pcbi.1014483.s010.pdf]

We thank the reviewers for their thorough and thoughtful feedback. Based upon reviewer feedback, we have made extensive improvements to the manuscript. These include:

- We have expanded our evaluations to include the pretrained ResNet model we initialized our models with, a pretrained microscopy foundation model OpenPhenom, and a DINOv3 model that we fine-tuned ourselves on our training datasets. We observe that although the DINOv3 model is superior to SimCLR and CLIP on our compound-replicate retrieval tasks, it still underperforms MICON, and is as similarly brittle in out-of-distribution settings as SimCLR and CLIP.
- We have added benchmarks on mechnism of action prediction using the BBBC036 dataset.
- We have made substantial writing and clarity updates to the manuscript. This includes removing the claim that our method is a counterfactual method and strengthening connections with prior works.

We hope that with these updates the manuscript is now suitable for publication in PLOS Computational Biology. Below, we respond to specific points from each reviewer in detail (reviewer comments in blue).

Reviewer 1:

- *Need for stronger baseline with same training and evaluation data:*
    - *Transfer Learning (from natural images) is a strong and simple baseline.*
    - *The current baseline (SimCLR) may not fully reflect state-of-the-art performance in self-supervised learning for morphological profiling. DINOv2 has shown superior performance in recent literature (see https://www.nature.com/articles/s41598-025-88825-4) and should thus be included as a stronger and more relevant baseline.*

We have expanded to add three new strong baselines – a ResNet model pretrained on natural images, the cell painting foundation mode OpenPhenom, and a DINOv3 model fine-tuned on our training datasets – to our analyses. We now include these in our compound-replicate retrieval results shown in Figures 4 and 5 (reproduced below) and Supplementary Tables 1-4. For the ResNet model, we use the same weights we fine-tune for our MICON, CLIP, and SimCLR models, to test that performance is due to self-supervised fine-tuning on JUMP CP, and not due to the base strength of transfer learning from natural images. For DINOv3, we initialized from weights for the ViT-B version of DINOv3 supplied by the official repository, and fine-tuned using our training datasets, reflecting a state-of-the-art self-supervised model fine-tuned on our datasets for fair comparison. All self-supervised models improve over the ResNet pre-trained models across our compound-replicate retrieval tasks, suggesting that self-supervised learning on microscopy datasets helps with performance. We show in Figures 4 and 5 and Supplementary Tables 1-4 that while DINOv3 models significantly outperform SimCLR and CLIP across compound-replicate retrieval tasks, MICON still outperforms DINOv3. Interestingly, the DINOv3 model still largely fails to generalize to out-of-distribution settings, and like our previous observation with SimCLR and CLIP, performs worse than CellProfiler on most tasks out-of-distribution even though it has far superior performance in-distribution. Again, this is not a trend observed for the MICON models. Surprisingly, OpenPhenom generally has poor performance, performing comparably

to or worse than CellProfiler across all evaluation settings, suggesting it may not transfer well.

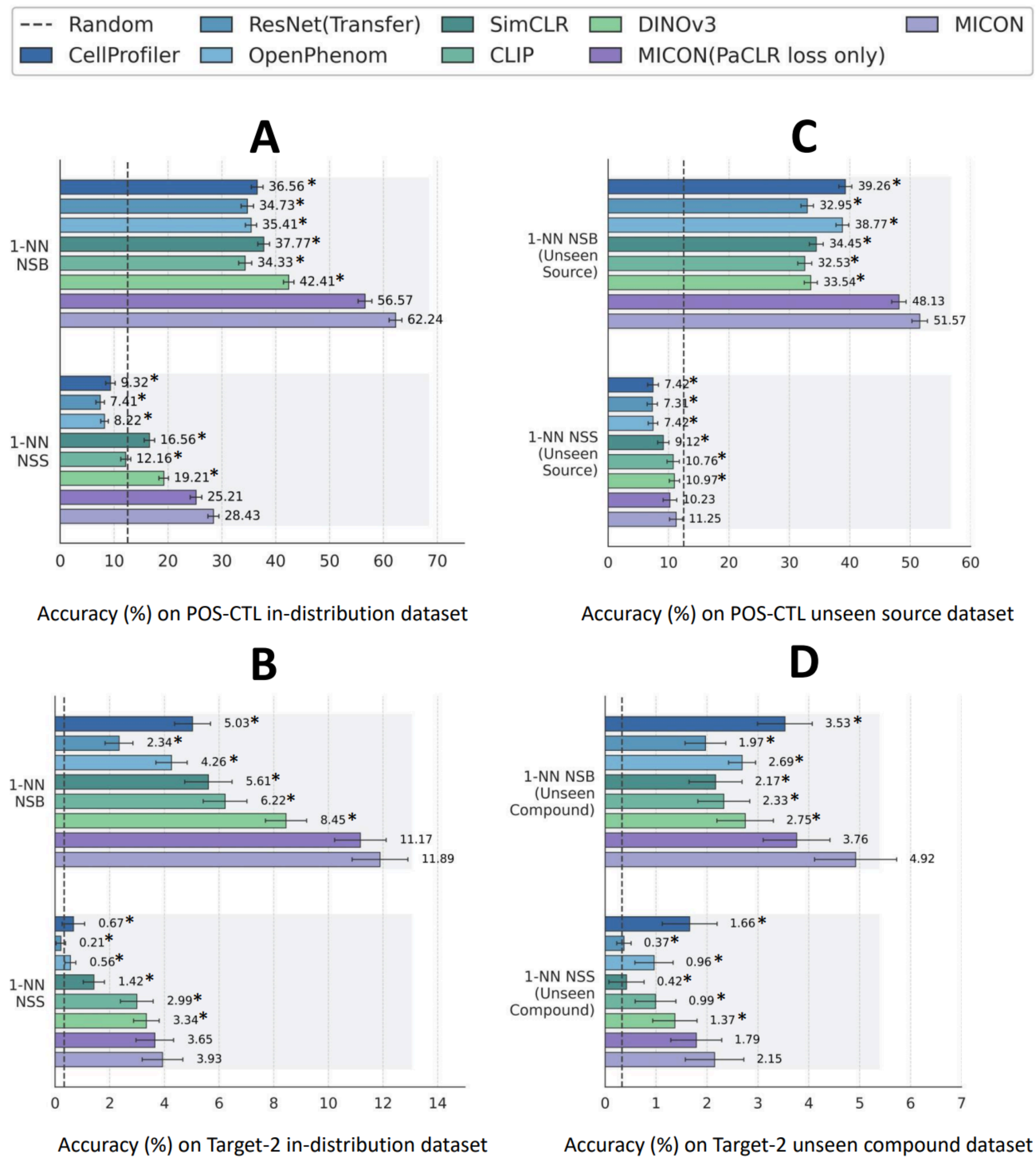

Figure 4: Compound-replicate retrieval accuracy for representation learning methods across evaluation settings. Retrieval accuracies on the (A) POS-CTL ID query dataset, (B) Target-2 ID query dataset, (C) POS-CTL OOD query dataset (unseen source), and (D) Target-2 OOD query dataset (unseen compounds). NSB designates retrieval of the nearest neighbor not in the same batch; NSS designates retrieval of the nearest neighbor not in the same source. n=3 models trained and evaluated with different dataset stratifications and different random seeds; the mean accuracy is labeled, and the error bars represent standard deviation Asterisk indicates that MICON significantly outperforms the baseline, defined as $p < 0.05$ on an unpaired one-tailed t-test with performance across the n=3 random seeds as trials.

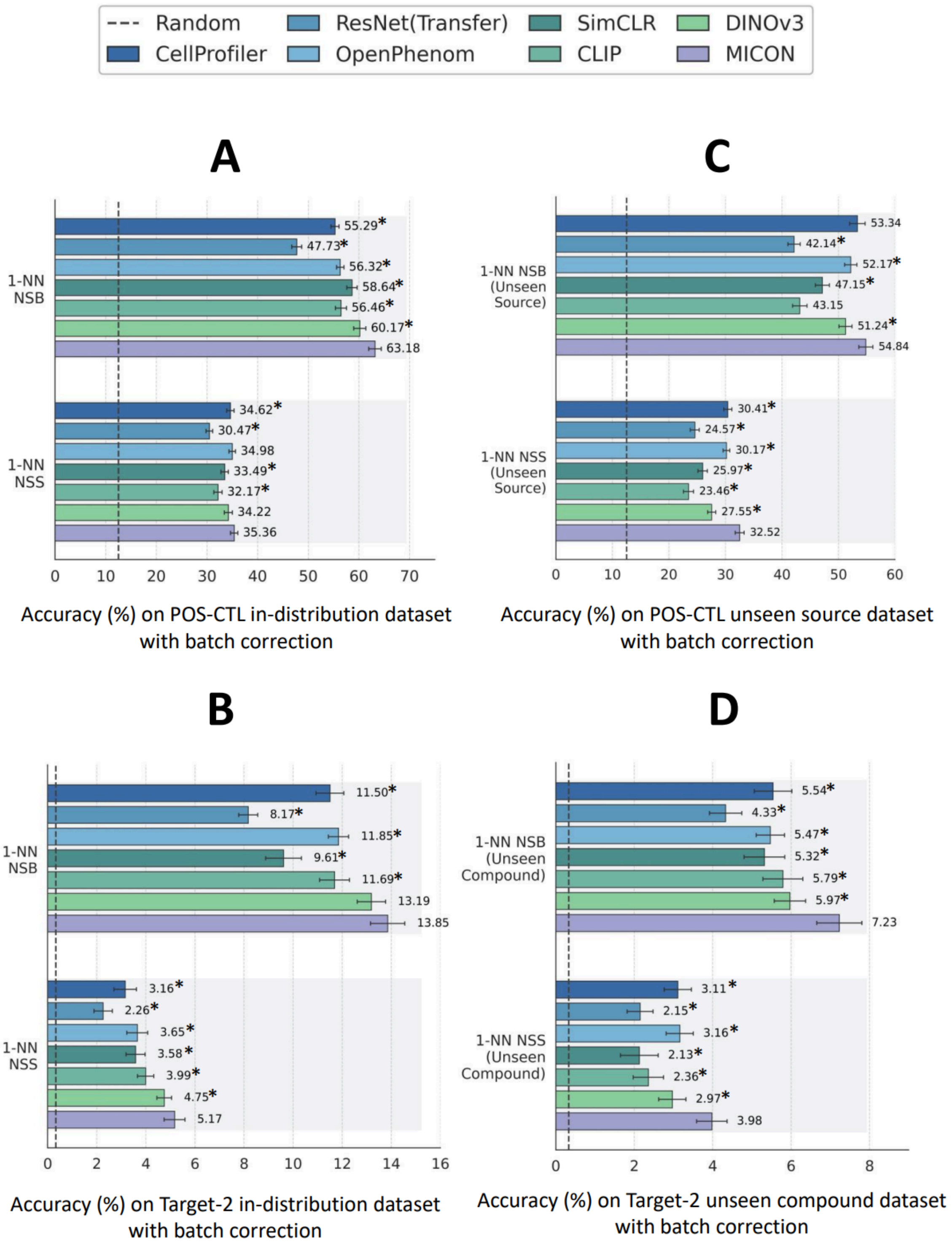

Figure 5: Compound-replicate retrieval accuracy for representation learning methods across evaluation settings, when microscopy batch effect correction is applied via spherizing. Retrieval accuracies on the (A) POS-CTL ID query dataset, (B) Target-2 ID query dataset, (C) POS-CTL OOD query dataset (unseen source), and (D) Target-2 OOD query dataset (unseen compounds). NSB designates retrieval of the nearest neighbor not in the same batch; NSS designates retrieval of the nearest neighbor not in the same source. n=3 models trained and evaluated with different dataset stratifications and different random seeds; the mean accuracy is labeled, and the error bars represent standard deviation. Asterisk indicates that MICON significantly outperforms the baseline, defined as $p < 0.05$ on an unpaired one-tailed t-test with performance across the n=3 random seeds as trials.

- *The evaluation could be extended to more task than replicate retrieval in order to assess the quality of the extracted biological signal:*
    - *Mechanism of Action classification (see https://www.nature.com/articles/s41467-024-45999-1)*
    - *Drug Target Interaction and known biological relationship recall (see: https://arxiv.org/pdf/2503.20158) highlighting the model's utility for practical biological understanding.*

We now include mechanism of action (MoA) enrichment benchmarks for the BBBC036 dataset in Section 3.5, with key results in Table 1 (reproduced below). In addition to the original fold-of-enrichment metric reported by Moshov *et al*., we also opted to report percent of compounds with a significant enrichment in retrieving compounds with the same mechanism of action. This is because we found that the original fold-of-enrichment metric was not robust to infinite odds-ratios, which occur when all compounds with the same MoA are ranked within the top 1% of retrieval (some compounds have few other compounds with the same MoA) - Moshov *et al*. impute the value with the size of the background set (1535) when this happens, but as this is much larger than the typical odds-ratio (7-11), this can lead to misleading differences between methods that behave similarly due to how much this imputation skews the average value.

Table 1: **Mechanism of action enrichment for the BBBC036 dataset.** We report average fold-of-enrichment with imputation (imputing the size of the background set as in [26]), and percent of significant compounds.

	Average Fold-of-Enrichment	Percent Significant Compounds
CellProfiler	25.62	9.20%
SimCLR	32.83	11.23%
CLIP	31.14	11.59%
DINOv3	31.23	11.23%
MICON	31.26	11.23%

Our finding is that unlike compound-replicate retrieval, all self-supervised methods (SimCLR, CLIP, DINOv3, and MICON) outperform CellProfiler, and all self-supervised methods perform similarly to each other. We believe this is a combination of two factors. First, MoA enrichment on the BBBC036 dataset has less challenging batch effects than our compound-replicate settings, and in the latter benchmarks, we showed that self-supervised methods other than MICON often excel in less extreme batch effect settings. Second, it is possible that these methods have saturated transferability from the CP JUMP datasets we trained on to BBBC036. Supporting this, DINOv3 also performs similarly to SimCLR and CLIP on this task, despite exhibiting significant improvements in our compound-replicate retrieval

settings. We show in the new Supplementary Figure A4 (reproduced below), overlap in compounds that are significantly enriched in retrieving compounds with the same MoA across methods. All self-supervised methods are significant for virtually the same compounds. Interestingly, CellProfiler retrieves a set of compounds distinct from all self-supervised methods, suggesting that self-supervised methods as a class are biased towards certain MoAs even with different pre-training tasks and architectures, at least when trained on the same dataset. We report full results in Section 3.5.

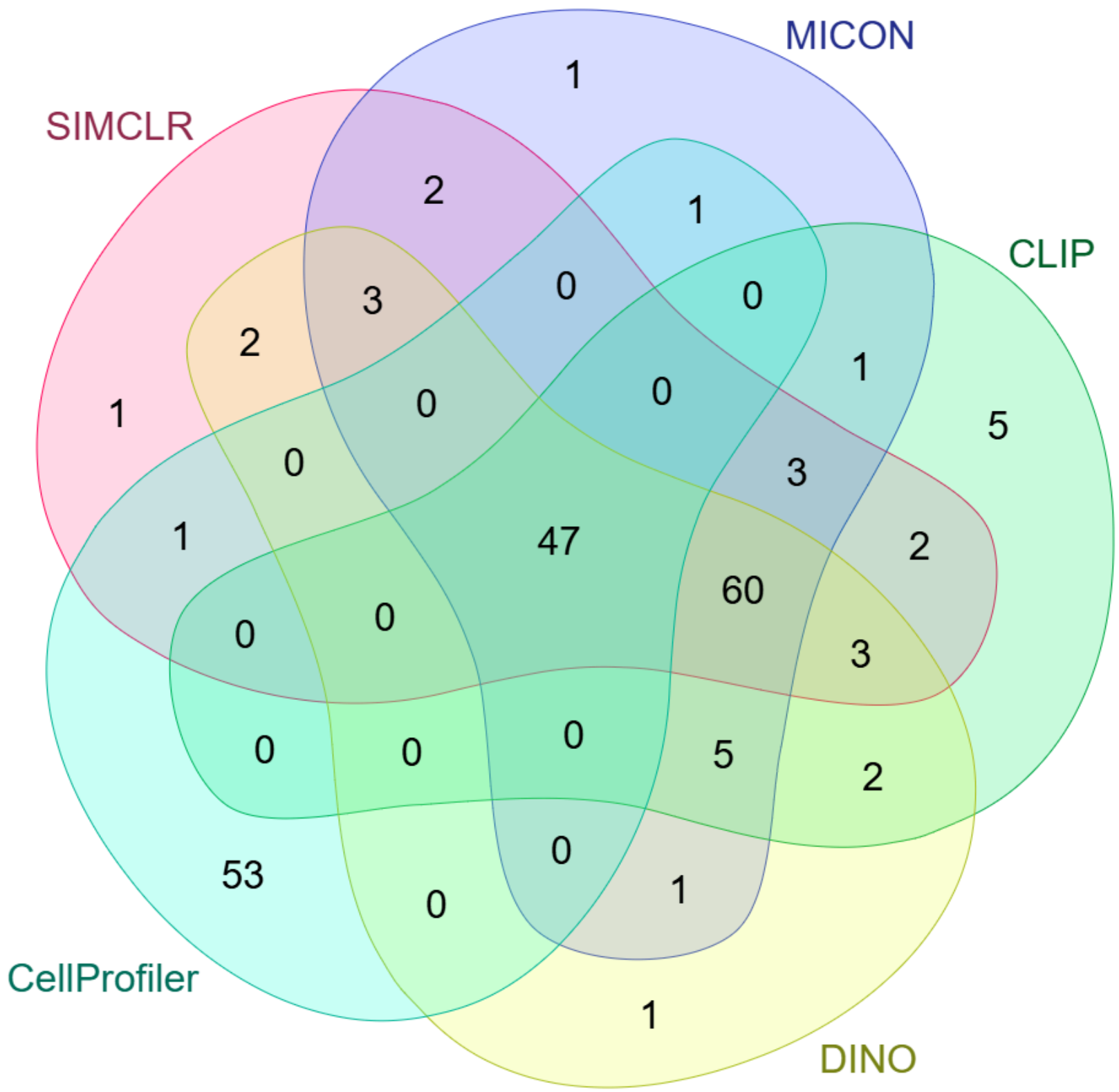

Fig Sup A4: Venn diagram showing overlap in compounds significantly enriched in retrieving compounds with the same mechanism of action for the BBBC036 dataset across methods.

We also experimented with known biological relationship recall tasks, but we found that existing datasets proposed for this task were confounded by batch effects (specifically, we observed that the plate arrangement for CRISPR knockout experiments in JUMP CP is held static across replicates, with closer-than-random proximity between genes of similar pathways). Hence, we elected not to include these results in this manuscript. A thorough exploration of these issues is beyond the scope of this manuscript, but we hope to document these in future work.

- *While synthesizing phenotypic effects for unseen compounds is highly valuable, a comparison to a more conventional and widely recognized model like CLIP (contrastive language-image pre-training) would provide a clearer benchmark for assessing innovation and performance advantages.*

While we deeply appreciate this suggestion, in practice, it is challenging to make a head-to-head comparison because molecular-image CLIP models (like CLOOME) cannot synthesize phenotypic effects for specific baseline untreated cells, and only output a single

fixed embedding for each compound. This is in contrast to our generative set-up, where we supply the closest negative control image to the perturbation we attempt to synthesize, under the assumption that baseline phenotype before treatment is similar due to shared batch effects.

Instead, we more explicitly disclose this as a limitation in our discussion:

> "Finally, while we demonstrate that MICON is capable of synthesizing the phenotypic effects of some unseen compounds as a consequence of its pretraining task, our evaluation was an initial proof-of-concept to determine if any predictive signal was present. It is unclear the effectiveness of this strategy compared to other proposals like CLIP-based strategies (Sanchez *et al*. 2023, Fradkin *et al*. 2024), and future work should do a comparative analysis."

Minor Points

- *As discussed by the authors, drug screening experiments typically include many compounds with minimal phenotypic impacts relative to negative controls. It would thus be valuable to specifically evaluate the model on such low-impact compounds to enhance real-world applicability.*

We agree this would be valuable. However, there are practical issues in curating an evaluation that reflects this: it is challenging to identify, in an unbiased way, compounds that have minimal phenotypic impacts, without risking selection of compounds that simply elicit no phenotypic response in cells. Instead of attempting to navigate this fine balance within our current work, we believe this is better explored in future directions. We have updated our discussion to more explicitly discuss this, as well as distinguish between impacts of our data curation on training versus evaluation:

> "Future work should explore if MICON stays robust when deployed on larger and less curated datasets. In this work, we focused on a small number of perturbations that were more likely to have strong phenotypic effects. However, drug screening experiments are usually systematic in nature and will thus include many compounds that elicit minimal phenotypic impact on cells relative to negative controls. **This impacts both training and evaluation. In training,** as MICON's PaCLR loss forces the model to distinguish compound-treated images from negative controls, it is not clear if scaling the number of compounds in the training dataset will adversely affect learning. Future work should analyze the trade-off between indiscriminately scaling the number of images and/or compounds versus downsampling datasets to just the stronger phenotypic effects. **In evaluation, we may not be evaluating the sensitivity of models to more subtle phenotypic changes. We did not attempt to do this in our work as curating compounds that have minimal but reproducible effects without risking selecting compounds that have no reproducible phenotypic impacts on cells is challenging, but we think this is crucial in future work.**"

- *Similarly, evaluating the model with varying numbers of images per compound—as suggested in the discussion—would strengthen the generalizability of results.*

While we agree this would be valuable, the current manuscript already involves training of many models on large datasets of 300-500k images: 5 models/baselines/ablations against 4 training data splits against 3 replicates for a total of 60 models. Adding varying numbers of images per compound would add another axis to this, greatly increasing the number of models trained, and is beyond the compute we can spare for this project. We note that this suggestion is effectively asking for a scaling law analysis, and while prior works of this nature have been immensely valuable, they are typically stand-alone manuscripts due to their onerous nature. Hence, this suggestion is better served in future projects.

- *Comparing methods that inherently correct batch effects (such as MICON) directly to methods without built-in batch correction (e.g., CellProfiler) without standard post-processing normalization may not provide a fair comparison.*
- *When batch-effect correction is applied, the chosen approach for CellProfiler features may not fully represent the current state-of-the-art methods (see https://www.nature.com/articles/s41467-024-50613-5).*

We agree with these points, and this was our rationale for the experiments in the current Figure 5 (originally Figure 6), where we assessed the interaction of batch effect correction using spherizing methods with all feature representations. The intention of this was to ensure that improvements from self-supervised learning could not be attributed to correction of trivial batch effects that would be removed by standard batch effect correction procedures post-hoc. Our results evidence that while this is not true for MICON (we still outperform CellProfiler even after spherizing), this is true for SimCLR and CLIP.

We have made this more explicit in section 3.4:

> "**As MICON learns to inherently correct for batch effects through its training set-up, which enforces similarity between images with the same perturbation across different batches, we next sought to confirm that performance improvements from MICON were not because of trivial correction of batch effects to the point that these commonly adopted standards for batch effect correction would equalize performance with baselines that did not inherently build in batch effect correction (e.g. CellProfiler).**"

Second, although we agree as demonstrated by the publication by Arevalo *et al*. 2024 the reviewer raises, there is evidence that batch effect correction methods pioneered in RNA-seq are effective for microscopy, the batch effect correction method that we employed is widely adopted and considered a standard for batch effect correction in imaging.

- *In the associated GitHub repository (github.com/microsoft/MICON), the provided link to "the official JUMP Cell Painting dataset" actually refers to the method proposed by Watkinson et al. (https://ieeexplore.ieee.org/document/10635200). This should be properly cited to avoid confusion.*

We thank the reviewer for pointing this out. We have updated the links to properly refer to the JUMP Cell Painting Consortium, and disambiguate from the work of Watkinson *et al*., for which we now provide both a link to the script and study.

- *There are a few typo:*
    - *Line 58: "ifmages" → "images"*
    - *Line 106: "(cpg0016, see Section)"—section reference is missing or incomplete (likely "Methods").*

We thank the reviewer for pointing these issues out. We have corrected them.

Reviewer 2:

Regarding evaluation of counterfactual representations. While MICON introduces a novel approach for generating counterfactual representations, its evaluation currently centers on nearest-neighbor retrieval accuracy. The authors should consider adding a more direct assessment of biological meaning. This assessment could involve comparing the phenotypic similarity distributions between generated counterfactuals and real perturbation images. Would it be possible to show a generated image? Visualization and quantitative comparison methods, such as UMAP projections, phenotypic clustering, or statistical/image reconstruction measures like Kolmogorov–Smirnov (KS) statistics, Earth Mover's Distance, PSNR, and LPIPs could further help determine how well these representations preserve biologically meaningful variation in relation to the other methods. Further, incorporating higher-level biological annotations (such as compound mechanisms of action (MoA) or known phenotypic groupings) would more clearly validate the relevance of the generated representations. Such analyses would not only demonstrate the realism of the counterfactuals but also their interpretability in a biological context.

We thank the reviewer for this suggestion, and agree that a more direct assessment of biological meaning would be useful for the manuscript overall. We note that we focused most of our analyses on representations of real images, and not the counterfactual generated images, as the scope of our work is more focused on using counterfactual generation as a pretraining task to improve image representation learning. In this context, the utility of counterfactual generation is if learning to do this improves our ability to represent real images, even if the performance of counterfactual generation itself ultimately remains low. While we do show one analysis that indicates that we can generate counterfactual representations for unseen compounds at any level of performance above random chance (which is exciting), the overall accuracy of our generation is low to the point that it is probably not useful, and hence, we didn't spend too much time on extensive validation.

But in the spirit of this suggestion, we did aim to better demonstrate that MICON's representations of real images are biologically meaningful. We now include mechanism of action classification benchmarks for the BBBC036 dataset in Section 3.5 and Table 1 (reproduced below). In addition to the original fold-of-enrichment metric reported by Moshov *et al.*, we also opted to report percent of compounds with a significant enrichment in retrieving compounds with the same mechanism of action. This is because we found that the original fold-of-enrichment metric was not robust to infinite odds-ratios, which occur when all compounds with the same MoA are ranked within the top 1% of retrieval (some compounds have few other compounds with the same MoA) - Moshov *et al*. impute the value with the size of the background set (1535) when this happens, but as this is much larger than the typical odds-ratio (7-11), this can lead to misleading differences between methods that behave similarly due to how much this imputation skews the average value.

Table 1: **Mechanism of action enrichment for the BBBC036 dataset.** We report average fold-of-enrichment with imputation (imputing the size of the background set as in [26]), and percent of significant compounds.

	Average Fold-of-Enrichment	Percent Significant Compounds
CellProfiler	25.62	9.20%
SimCLR	32.83	11.23%
CLIP	31.14	11.59%
DINOv3	31.23	11.23%
MICON	31.26	11.23%

Our finding is that unlike compound-replicate retrieval, all self-supervised methods (SimCLR, CLIP, DINOv3, and MICON) outperform CellProfiler, and all self-supervised methods perform similarly to each other. We believe this is a combination of two factors. First, MoA enrichment on the BBBC036 dataset has less challenging batch effects than our compound-replicate settings, and in the latter benchmarks, we showed that self-supervised methods other than MICON often excel in less extreme batch effect settings. Second, it is possible that these methods have saturated transferability from the CP JUMP datasets we trained on to BBBC036. Supporting this, DINOv3 also performs similarly to SimCLR and CLIP on this task, despite exhibiting significant improvements in our compound-replicate retrieval settings. We show in the new Supplementary Figure 4 (reproduced below), overlap in compounds that are significantly enriched in retrieving compounds with the same MoA across methods. All self-supervised methods are significant for virtually the same compounds. Interestingly, CellProfiler retrieves a set of compounds distinct from all self-supervised methods, suggesting that self-supervised methods as a class are biased towards certain MoAs even with different pre-training tasks and architectures, at least when trained on the same dataset. We report full results in Section 3.5.

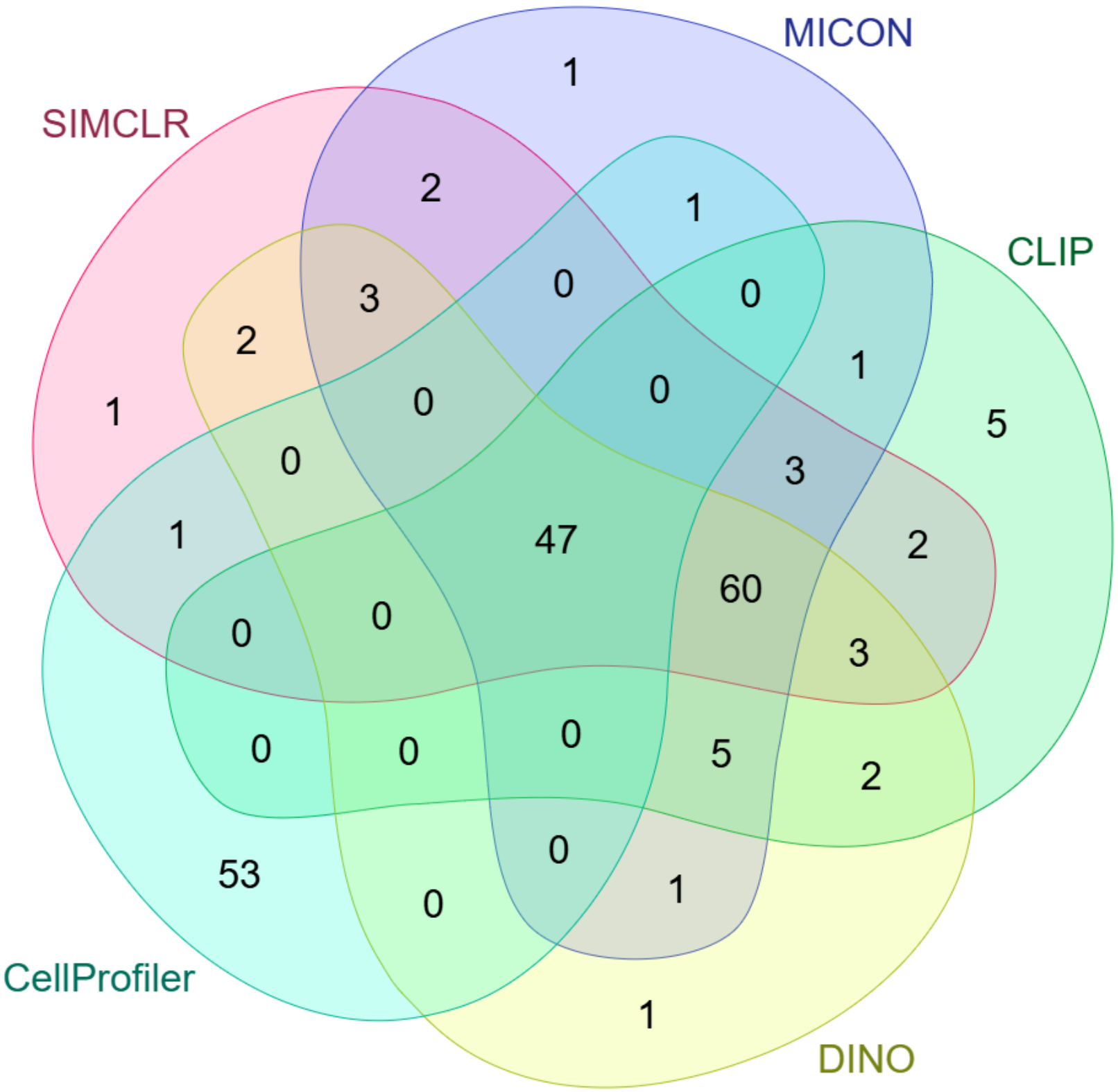

Fig Sup 4: Venn diagram showing overlap in compounds significantly enriched in retrieving compounds with the same mechanism of action for the BBBC036 dataset across methods.

Benchmarking other approaches. The manuscript would benefit from a broader comparative evaluation against recent state-of-the-art deep learning frameworks. Notably, adding SPACe as an additional baseline for hand-crafted features would be particularly informative, given its strong performance on JUMP-CP datasets, and outperformance of CellProfiler features. Additionally, multimodal methods such as CLOOME and MolPhenix integrate compound structure with image-based data in ways that are conceptually related to MICON's approach. While direct comparisons may be complicated by differences in architecture or pretraining strategies, a more detailed discussion, or even a proxy evaluation using similar training objectives, would clarify the distinct advantages of MICON's causal integration strategy. Such analysis would strengthen the empirical foundation of the work and better contextualize its contributions within the evolving landscape of multimodal representation learning. Refocusing the methodologies presented here as a benchmark and adding these methods would add a lot of oomph.

In response to this suggestion (and suggestions by other reviewers), we have substantially expanded our benchmarking suite. We now additionally benchmark:

1. A ResNet model, pretrained on natural images. This is the pretrained initialization for all of our models, and validates that self-supervised learning on microscopy images improves on what could potentially be a strong starting point learned from natural images.
2. OpenPhenom, a cell painting foundation model trained using masked autoencoding.
3. DINOv3, a state-of-the-art self-supervised learning model, fine-tuned on our training datasets.

We avoided CLOOME and MolPhenix specifically because of our evaluation set-up, which holds out some chemical compounds and tests for generality to unseen compounds, because these models have already seen these compounds during pre-training. However, we do note that what we call CLIP (both in the original manuscript and the updated version) actually is the method underlying both CLOOME and MolPhenix; our CLIP baseline is retrained on our training datasets and with a standardized architecture to enable head-to-head comparison with MICON. We hope this is clear in our methods:

> "A CLIP-based approach for multimodal learning between microscopy images and chemical compounds was previously proposed by CLOOME and extended in MolPhenix. As these works use different architectures, datasets, and pre-trained models, they do not provide appropriate baseline controls to MICON. We train a CLIP baseline model using the same dataset and encoder architectures as MICON to enable direct comparison of the method itself, instead of transferring these models, which could result in disparities in performance from these other factors. Thus, we refer to the resulting baseline model as "CLIP.""

Minor comments

1. *Additional detail is needed regarding the final loss formulation. Specifically, it is unclear whether the PAC and PaCLR loss are equally weighted in the overall objective. Were alternative weighting schemes considered or tuned during development? Would there be any benefit to parameterizing the contribution of each component? Clarifying this aspect would help readers better understand the relative contribution of each component to the optimization process and how the model balances learning from real versus counterfactual examples.*

In our work, we weighted the real and predicted PaCLR losses equally. This was the simplest default, and not a parameter that we explored in this work, but the reviewers are correct that different weight configurations could make a difference for learning.

We have made this more clear in our methods:

> "The final loss for MICON combines the real and predicted PaCLR objectives. **We weight these loss components equally**"

And incorporated this into our discussion:

> "**Finally, a more thorough exploration of hyperparameters could further advance this work. In our experiments, we equally weighted the real versus predicted PaCLR losses. We demonstrated that although we have some signal in synthesizing the phenotypic effects of unseen compounds, our performance is weak, and one possibility is that we dedicate too little of our loss to generating predictions: increasing the weight on this loss, either throughout training or during later epochs, could improve performance in generating synthetic image representations.**"

2. *There were a few typos. Figure 1 legend: "pertubation" should be corrected to "perturbation." Line 54: "ifmages" is a typo; should be "images." Line 90:*

We thank the reviewers for raising this to our attention. We have fixed these typos.

3. *"pertubation-aware" should be corrected to "perturbation-aware." Supplementary Figure 3: "magneta" should be corrected to "magenta."*

We thank the reviewers for raising this to our attention. We have fixed these typos.

4. *Figure 1 is very clear and effective, but we wonder why the matrix is duplicated in panel C, does this have meaning?*

Yes - Panel B refers to our loss on real images exclusively, while Panel C refers to our loss for predicted image representations. A key distinction is that we use negative controls when learning on real images, but never when predicting image representations (as all predicted image representations are perturbed) - negative controls are shown in orange, and they are absent in Panel C. We have tweaked some headings in the figure to make this clearer (reproduced below).

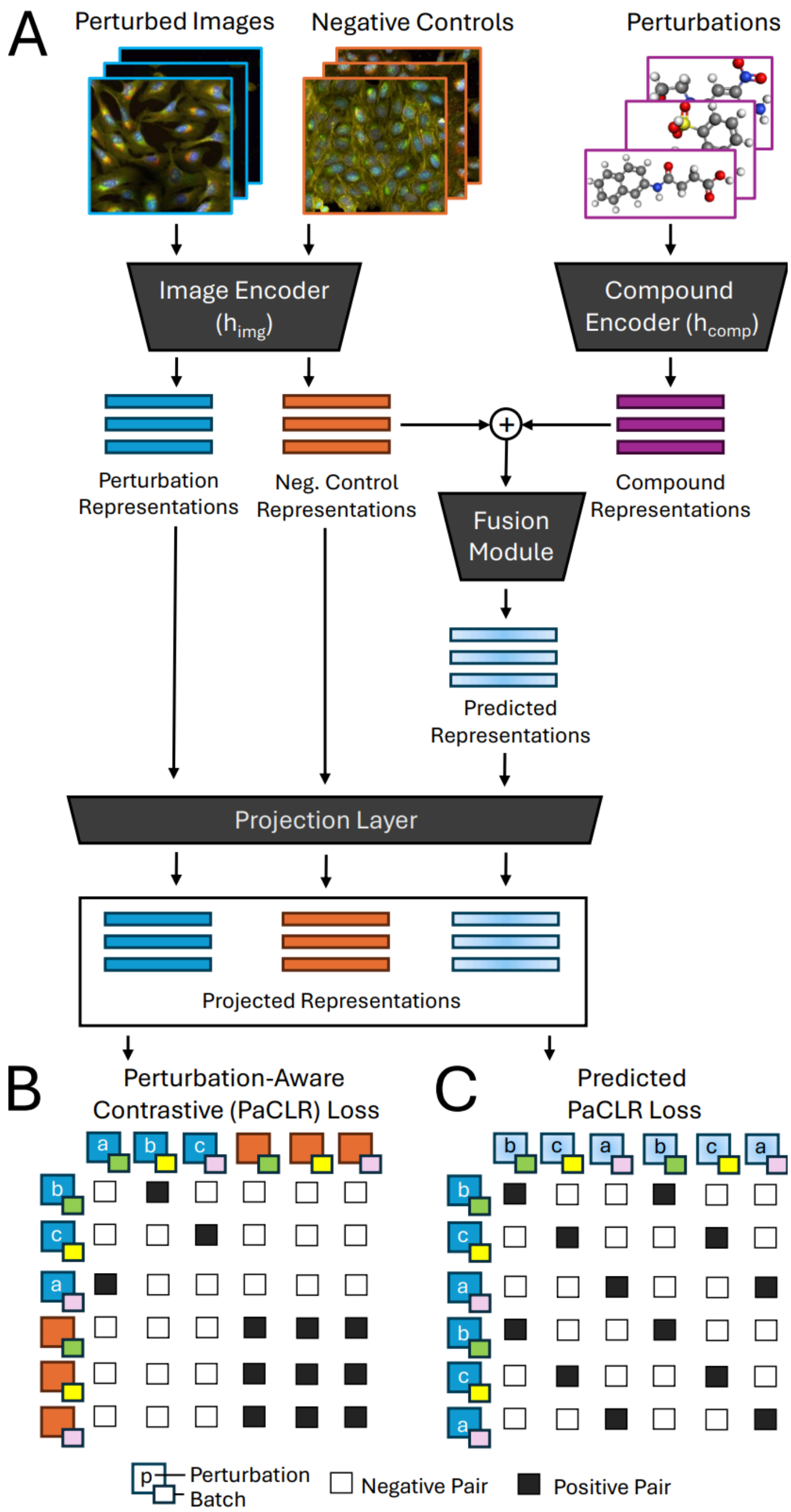

Figure 1: Summary of the MICON framework. (A) An overview of our model. Compound perturbed (blue) images and negative control (orange) images are transformed into representations by an image encoder, while chemical compounds (purple) are transformed into representations by a compound encoder. A fusion module accepts pairs of negative control representations and compound representations and outputs predicted representations (light blue). All representations are passed through a projection layer to form the final projected representations. (B) Summary of the perturbation-aware contrastive (PAC) loss. Perturbed images (blue) and negative controls (orange) from the same batches as the perturbed images (batches denoted by small colored boxes) are sampled, with at least two images for each perturbation. Images treated with the same perturbation (a, b, or c) serve as positive pairs (black squares) for the PaCLR loss. (C) Summary of the predicted PaCLR loss. Perturbation representations (blue) and predicted representations (light blue) using the same compound (a, b, or c) serve as positive pairs (black squares) for the predicted PaCLR loss.

5. *Reference to a specific section missing in line 106, also lines 153, 235, 282, 388*

We thank the reviewers for raising this to our attention. We have fixed this.

6. *It would be helpful to justify choice of cosine distance in simCLR loss function on line 128*

We have made it clearer that this is consistent with previous contrastive learning works:

"(in our work, the cosine distance, **consistent with standards established by SimCLR**)"

7. *It would be helpful to clarify if the method treats FOVs from the same image in the same way as FOVs from different images (Line 132-134)*

We now clarify this in Section 2.2.1 about the dataset. We make it clearer that we consider "wells" to be "images" for the purposes of our sampling, which means that FOVs from the same well are never sampled within the same training batch:

> "**Note that for our loss, we consider a well to be an image x, and sample a random FOV for the well. This has the impact that no FOVs from the same well are ever within the same batch of data during training of our method.**"

8. *The authors present a wonderfully detailed and accurate description of JUMP.*

We thank the reviewers for this compliment! In response to Reviewer 3, we have further improved this section by being more precise about the parts of JUMP CP that are employed for our work. While we still provide an overview of the data collection procedure, which we think is essential for readers unfamiliar with the field in understanding how our evaluation set-up tests for robustness to batch effects, we now focus more precisely on just the subsets of JUMP we curated data from (instead of the entire data collection efforts) and disclose that our datasets are a small subset of the total data collection:

> "**We curate several kinds of plates and wells from the cpg0016 dataset for training and evaluating our models. First, we curate positive control wells (abbreviated as POS-CTL in this work) from the entire dataset; these reflect 8 compounds selected to have the most distinct morphological changes in a pilot study. Most plates in cpg0016 have 4 well replicates of each of these 8 positive controls. Second, we curate JUMP-Target-2-Compound plates (abbreviated as Target-2 in this work), which image 301 compounds selected as likely to induce phenotypic effects in cells and are measured in replicate by every source. Third, we curate a subset of 184 compounds from the Compound plates of cpg0016 (see Section 2.2.2 for further details). Finally, every plate includes negative control wells treated with Dimethyl Sulfoxide (DMSO), the solvent used for all compound perturbations, which acts as a control where no compound is dissolved in DMSO. We curate negative controls from all plates from which we sampled POS-CTL, Target-2, and Compound wells. Together, our dataset comprises 87,282 of the 1,096,069 wells in cpg0016**."

9. *The data splitting procedures and evaluation strategies for them are elegantly designed. However, I'm wondering if the 184 compounds unseen during training*

could be split even further based on target or mechanism of action. For example, It would be more impressive if the model would work on an entirely new MOA.

The 184 compounds in our split actually share no overlap in mechanism of action with the Target-2 compounds! We make this clearer in the manuscript:

> “We then nominated compounds in the top 10% of greatest distances for at least four sources, yielding a total of 184 compounds. **All of these compounds have disjoint mechanisms of action (MoAs) with the Target-2 compounds**.”

10. *We did not anticipate seeing accuracy so low! It would be helpful to discuss this sooner, in the abstract (or even in the title). It would also be helpful to discuss in what cases this approach would be useful. Similarly, maybe reporting top3 or top5 matching would be helpful?*

We believe this is due to the very strong degree of batch effects, particularly across sources. To demonstrate this, we have added Supplementary Figure 1 (reproduced below), which shows the cosine similarity between averaged CellProfiler features across all negative controls for each batch. This shows that even as the cosine similarity is close to 1.0 for negative controls across batches, between certain batches, the cosine similarity can be nearly 0, demonstrating how aggressive batch effects can be in this dataset. We hope this figure helps contextualize why compound-replicate retrieval accuracy is so low especially in the not-in-same-source tasks.

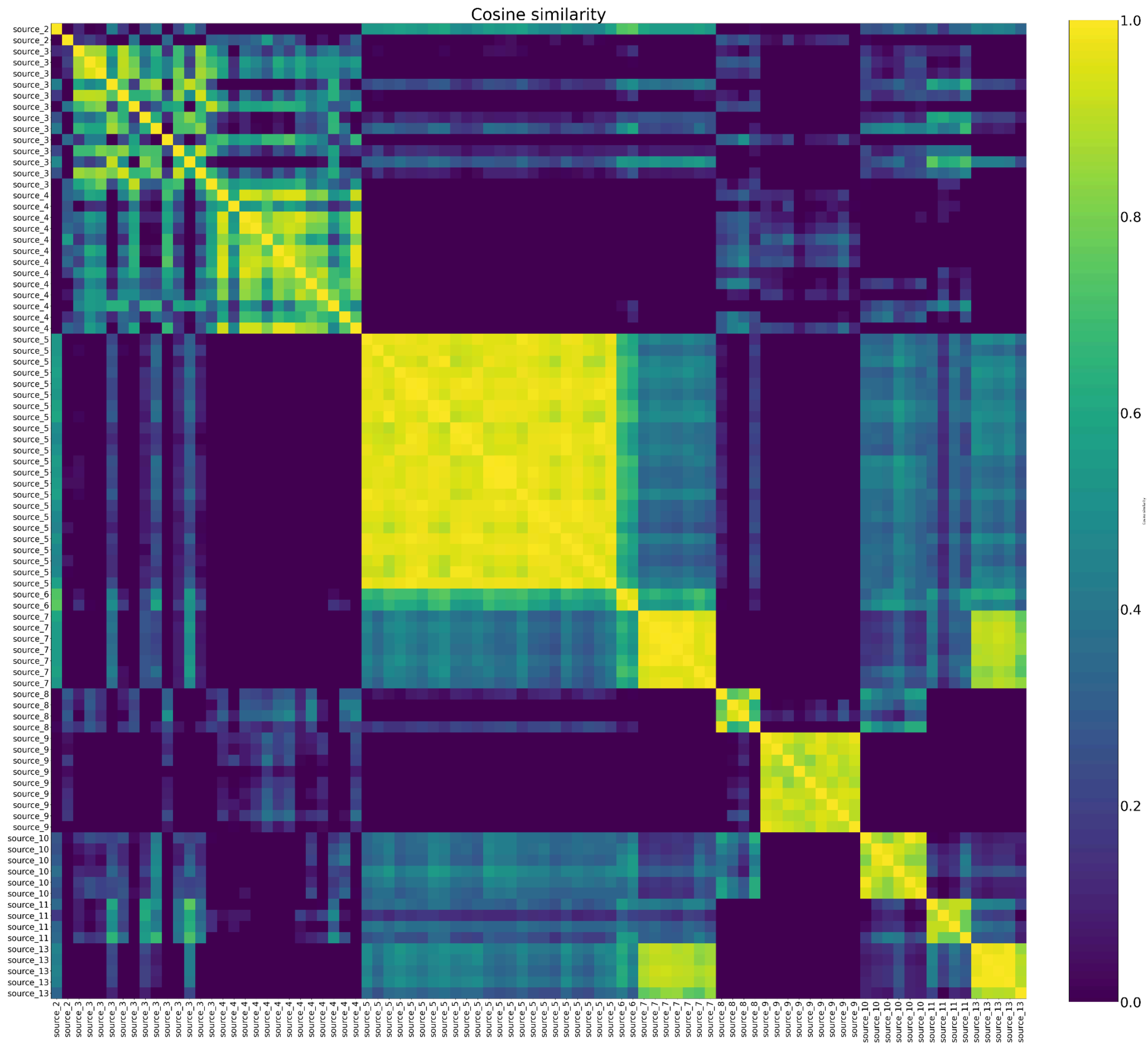

Fig Sup 1: Heat map showing cosine similarity between averaged CellProfiler features across negative control wells for each batch. Batches (x and y-axes) are labeled by their source in CP JUMP.

Additionally, we have added Supplementary Tables 1-4 with Top 3 and Top 5 matching, and incorporated this into the opening paragraph of our Results section:

> **"We report 1-nearest neighbor matching results in the main manuscript, but top-3 and top-5 results are available as Supplementary Tables Sup 1, Sup 2, Sup 3, and Sup 4."**

We find that the ranking of models by performance is generally preserved when looking at Top 3 and Top 5 matching compared to 1-nearest neighbor.

11. *It is refreshing to see Figure 6 results and interpretation regarding the comparison to CellProfiler after batch effect correction seeing minimal performance improvements. Kudos!*

We are thankful that the reviewers appreciate this result! As we also noted in our response to Reviewer 1, the intention of this was to ensure that improvements from self-supervised

learning could not be attributed to correction of trivial batch effects that would be removed by standard batch effect correction procedures post-hoc. We are pleased that MICON still continues to consistently outperform CellProfiler after batch effect correction, whereas the same cannot be said true for other self-supervised methods.

12. *The authors include a dropbox link for model weights and representations. This is brittle and liable to 404 at any time. We recommend that the authors instead deposit this data in a third-party resource (e.g., figshare), which will perpetuate and generate a DOI.*

We thank the reviewers for raising this issue. We have uploaded our data to HuggingFace and replaced the Dropbox link with this.

Reviewer 3:

Comments

1. Causal integration

- *The authors claim that their framework is a causal representation learning framework for morphological profiling (lines 60-62). This claim is not accurate in the context of recent machine learning work that investigates causal representation learning from the perspective of causal inference, causal structural models, and Pearl's ladder of causation (see e.g., [A]). Given that the proposed method is not aligned with these efforts, it is recommended to drop such terminology from the paper.*
- *The causal model in Figure 2 is similar to, but not consistent with previous literature in the field. For instance, [D] and [E] present and formalize an identical graph, which differs from the variables and causal relationships presented in this manuscript. Why is that difference? This manuscript does not justify the choice, does not explain the differences, and ignores these previous efforts. This suggests that the adoption of causal terminology is rather rushed and not considered carefully in the context of existing literature. If a causal graph is needed, it is recommended that the authors adopt the previously existing model or formalize a new one based on evolving needs for the proposed model (which does not seem to be the case).*
- *Regarding counterfactual representations, the model is not really performing counterfactual inference to deduce these representations. The model design is more consistent with a conditional generative model, with chemical structures and image features as a prompt, and new feature representations as a response. This is similar to the design of contemporary generative models, such as language models, where given a prompt, consistent outputs are generated, without the responses being necessarily counterfactuals. In fact, it is believed that the outputs are the result of interpolations of knowledge possessed by the models instead of counterfactual reasoning. In the proposed approach, batches and other variables of the causal model are not explicitly considered in the process. Counterfactual computation is a specific branch of active research in statistics and machine learning and the proposed model is not consistent with such literature. It is recommended to drop the counterfactual terminology and present it as an estimation of treated representations instead.*

- *The proposed methodology is clearly inspired by prior knowledge about the experimental design of perturbation experiments, serving as an effective prior to improve performance. However, the current terminology around causal representation learning and counterfactuals is confusing, and does not accurately reflect what the methodology is about. In practice, the proposed strategy is a sampling strategy that carefully selects minibatches with positive and negative examples that make sense for the problem. The method does not incorporate explicit inductive biases in the architectures or probabilistic mechanisms of the model, nor does it implement any type of causal inference in practice. Making the link with causal representation learning and counterfactuals is a stretch of terminology that does not benefit either the manuscript or the community.*

We thank the reviewer for their detailed explanation of why causal and counterfactual terminology is not appropriate for our method. In response, we have extensively edited the manuscript to remove such claims. We instead now claim that our method integrates chemical structures as treatments, as reflected in the new title, "Integrating chemical structures as treatments improves representations of microscopy images for morphological profiling." Although we agree our method is consistent with conditional generation methods, we avoid the term generation as we felt this term may imply *image* generation, as opposed to generation of representations, and throughout the manuscript we are careful to use terminology of synthesis or prediction instead, which we feel avoids confusion. Along with other changes, this means that the original Figure 2 showing the causal graph has now been removed.

- *The use of experimental metadata has been extensively explored in previous literature too. For instance, [B] and [C] extend self-supervised methods to leverage images of cells with the same treatment. These ideas are also connected with previous literature that use weakly supervised learning for representation learning [D]. It is recommended that the authors acknowledge these connections and explain the differences, as the current manuscript seems to claim that using such metadata is a novel idea.*

We have edited the Methods section to be explicit that the idea of using metadata in pretraining has been explored in previous works, and to explain points of difference in our implementation:

> "**MICON's integration of metadata about perturbation into representation learning is an idea also explored by several previous works, but our implementation of this idea has several key differences. Compared to Set-DINO [39] and WS-DINO [11], we use images treated with the same perturbation as positive pairs for contrastive learning, instead of enforcing similarity in a student-teacher set-up. Compared to weakly supervised proposals [5,26], we use metadata about perturbations for sampling pairs, rather than as predictive targets. Finally, we note that unlike many other prior works which only use perturbed images [11,34], we explicitly include negative control images.** We incorporate negative controls because, due to their lack of perturbation treatment, they primarily represent apparent phenotypic changes caused by batch effects and thus enable disentanglement of batch effects from perturbation effects."

[39] - Yao *et al*. 2024
[11] - Cross *et al*. 2022
[5] - Caicedo *et al*. 2018
[26] - Moshkov *et al*. 2024
[34] - Sanchez *et al*. 2023

We hope these changes better position MICON's base representation learning method, and make it clear the use of experimental metadata is not a novel idea, although we make key adaptations to accommodate this in a contrastive learning framework and with the use of negative controls (both of which our chemical compound fusion model build upon).

2. Experimental evaluation

- *The experimental results presented in the paper are extremely interesting, as they demonstrate that careful training of models can make a difference in robustness to batch effects. The manuscript does an excellent job at testing out of distribution performance by leaving batches and sources out.*
- *It is recommended to report what is the performance of ResNet features only without the proposed model. This can establish a baseline to estimate the contribution of the components of the model. Additional similar ablations are also recommended.*

We thank the reviewer for this suggestion. Integrating these suggestions with those from the other reviewers, we have substantially expanded our benchmarking suite. We now additionally benchmark:

1. A ResNet model, pretrained on natural images. This is the pretrained initialization for all of our models.
2. OpenPhenom, a cell painting foundation model trained using masked autoencoding.
3. DINOv3, a state-of-the-art self-supervised learning model, fine-tuned on our training datasets.

Addressing the reviewer's point, the first benchmark validates that self-supervised learning on microscopy images improves on what could potentially be a strong starting point learned from natural images. This model underperforms all self-supervised methods across our compound-replicate retrieval settings, suggesting that the self-supervised learning methods that we benchmark all improve performance over baseline initialization.

- *The central assumption of the manuscript is that same-compound retrieval is a good proxy for representation learning evaluation. However, this type of evaluation only checks replicability of the image signal, but not its biological significance. In other words, compounds that have similar effects may be represented with perfect clusters of replicates, but may be far apart in the feature space even when they are supposed to be related. This type of biological outcome evaluation is typically implemented with mechanism of action prediction tasks, for instance. It is recommended to implement such metrics in the analysis to confirm that the model is not just optimizing clusters of replicates, but rather finding meaningful connections between treatments.*
- *The evaluation with biologically relevant labels (e.g., mechanism of action) may require additional data beyond the strong treatments selected for this study. This is*

also a potential limitation of the manuscript that can be addressed by looking at increasingly more complex queries and references.

We agree with the reviewer that additional tasks would help assess if the representation learning learns biological significance, and thank them for this suggestion. We now include mechanism of action classification benchmarks for the BBBC036 dataset in Section 3.5 and Table 1 (table reproduced below). In addition to the original fold-of-enrichment metric reported by Moshov *et al*., we also opted to report percent of compounds with a significant enrichment in retrieving compounds with the same mechanism of action. This is because we found that the original fold-of-enrichment metric was not robust to infinite odds-ratios, which occur when all compounds with the same MoA are ranked within the top 1% of retrieval (some compounds have few other compounds with the same MoA) - Moshov *et al*. impute the value with the size of the background set (1535) when this happens, but as this is much larger than the typical odds-ratio (7-11), this can lead to misleading differences between methods that behave similarly due to how much this imputation skews the average value.

Table 1: **Mechanism of action enrichment for the BBBC036 dataset.** We report average fold-of-enrichment with imputation (imputing the size of the background set as in [26]), and percent of significant compounds.

	Average Fold-of-Enrichment	Percent Significant Compounds
CellProfiler	25.62	9.20%
SimCLR	32.83	11.23%
CLIP	31.14	11.59%
DINOv3	31.23	11.23%
MICON	31.26	11.23%

Our finding is that unlike compound-replicate retrieval, all self-supervised methods (SimCLR, CLIP, DINOv3, and MICON) outperform CellProfiler, and all self-supervised methods perform similarly to each other. We believe this is a combination of two factors. First, MoA enrichment on the BBBC036 dataset has less challenging batch effects than our compound-replicate settings, and in the latter benchmarks, we showed that self-supervised methods other than MICON often excel in less extreme batch effect settings. Second, it is possible that these methods have saturated transferability from the CP JUMP datasets we trained on to BBBC036. Supporting this, DINOv3 also performs similarly to SimCLR and CLIP on this task, despite exhibiting significant improvements in our compound-replicate retrieval settings. We show in the new Supplementary Figure 4 (reproduced below), overlap in compounds that are significantly enriched in retrieving compounds with the same MoA across methods. All self-supervised methods are significant for virtually the same compounds. Interestingly, CellProfiler retrieves a set of compounds distinct from all self-supervised methods, suggesting that self-supervised methods as a class are biased towards certain MoAs even with different pre-training tasks and architectures, at least when trained on the same dataset. We report full results in Section 3.5.

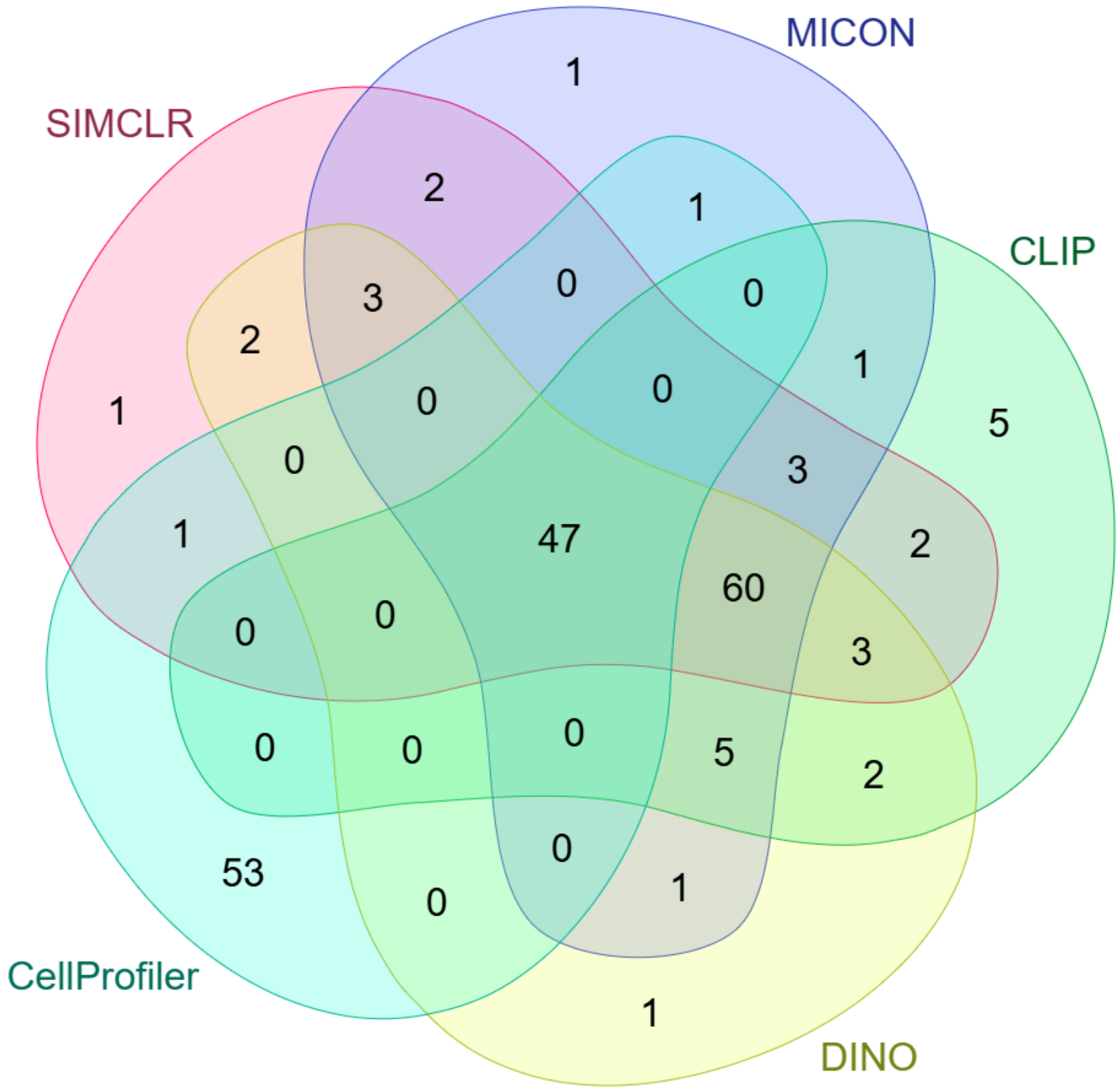

Figure A4: Venn diagram showing overlap in compounds significantly enriched in retrieving compounds with the same mechanism of action for the BBBC036 dataset across methods.

- *To back-up the claims that the synthetic representations can simulate treatment effects, it is important to evaluate biologically relevant tasks, such as mechanism of action prediction (see previous point). It is unclear whether the model can actually simulate treatments or just mimic representations that are expected in a cluster of replicates. Qualitative evaluations can be helpful in addition to the metrics, to illustrate that compounds with similar MoAs actually share similar representations.*

As we are focused more upon evaluating the claim that synthetic representations can be used to improve representations of real images in this study, our analysis of synthetic representations is meant to be a lightweight proof-of-concept (i.e. because our models have been pre-trained to do this, we can use them to generate synthetic representations, and it is interesting that we can synthesize even unseen compounds at some level of performance above random). Realistically, we observe that the performance of synthesis of unseen compounds is fairly low, so this capability of our model is likely not useful for any serious downstream applications, yet. Further model development would be required, and hence we think that more extensive evaluations of performance of the MICON model in generation of synthetic images or representations would not be appropriate at this stage.

We have sought to make this more clear both in the interpretation of these results:

> "These results suggest that MICON's predicted generations capture phenotypic effects of compounds seen in training, with some ability to capture the phenotypic impact of completely unseen compounds. **However, we caveat that overall performance is low especially for unseen compounds, suggesting that MICON is likely not useful for prediction yet in its current state.**"

And in the discussion, which also caveats the limited extent of our evaluation of synthetic representations:

> **"Third, while we demonstrate that MICON is capable of synthesizing the phenotypic effects of some unseen compounds as a consequence of its pretraining task, our evaluation was an initial proof-of-concept to determine if any predictive signal was present. It is unclear the effectiveness of this strategy compared to other proposals like CLIP-based strategies [34, 12], and future work should do a comparative analysis. Finally, a more thorough exploration of hyperparameters could further advance this work: in our experiments, we equally weighted the real versus predicted PaCLR losses. We demonstrated that although we have some signal in synthesizing the phenotypic effects of unseen compounds, our performance is weak, and one possibility is that we dedicate too little of our loss to generating predictions: increasing the weight on this loss, either throughout training or during later epochs, may enhance performance in producing synthetic image representations**."
>
> [34] - Sanchez *et al*. 2023
> [12] - Fradkin *et al*. 2024

- *The manuscript claims that their evaluation is the most rigorous. However, previous work [F] has proposed more extensive evaluation frameworks on the same dataset, considering generalization scenarios and biological tasks that are left out of this manuscript. Please, consider adopting those metrics, or adjusting the language to acknowledge these other efforts.*

We thank the reviewer for raising this issue, and it was not our intention to diminish other works. The contributions of Arevalo *et al*. are substantial in this field. In contrast to this work, which focuses on batch effect correction methods, our work focuses on benchmarking representation learning methods, and we have sought to make this complementary contribution more clear in our Discussion:

> "**Our evaluation also sheds novel insights into representation learning for cell painting.** Taking advantage of the large-scale and multi-source nature of the JUMP-CP dataset, we designed evaluation splits that allow us to assess if morphological profiling methods can maintain performance when comparing images from different data-generating sources and when sources or compounds are unseen during representation learning (Figure 2, 3). **While evaluations that stratify across sources have been designed to benchmark batch effect correction methods [2], our study systematically benchmarks different representation learning methods in a controlled way, using the same architectures and training data. Previous evaluations of representation learning typically only use datasets collected by a single source, and do not stratify compounds/sources such that some are unseen during training** [36,34,12]. Here, our benchmarks expose that some representation learning methods previously reported to be effective for morphological profiling may be overfitting and thus overestimating their performance.

For example, while SimCLR outperforms CellProfiler features when representing seen compounds, it underperforms CellProfiler on compounds unseen during training. In contrast, MICON is robust and demonstrates consistent improvements over both CellProfiler and baseline representation learning methods across all scenarios evaluated, even when compounds are unseen in training and even when considering more challenging batch effects like differing imaging sources."

[2] - Arevalo *et al*. 2024

3. Writing and presentation.

- *The description of the dataset presents details of the full JUMP dataset, which is not really used in the manuscript. This is unnecessary and misleading. The JUMP dataset can be simply cited and the subsets described, without inflating expectations about what the model was tested on.*

We thank the reviewer for raising the point that our extended description of JUMP CP may inflate expectations for readers. Our initial intent in writing this was to ensure that readers without a background in high-throughput microscopy understood how the underlying data generating process creates batch effects, including what microscopy batches, plates, wells, and fields of view are, as this is essential to understanding our method and evaluation. For this reason, we retain these details in the revised manuscript. However, we do re-write this section to focus more precisely on the subsets of data we curate images from, and also explicitly disclose how many wells in the total dataset are used for training and evaluation:

"**We curate several kinds of plates and wells from the cpg0016 dataset for training and evaluating our models. First, we curate positive control wells (abbreviated as POS-CTL in this work) from the entire dataset, 8 compounds selected to have the most distinct morphological changes in a pilot study. Most plates in cpg0016 have 4 well replicates of each of these 8 positive controls. Second, we curate JUMP-Target-2-Compound plates (abbreviated as Target-2 in this work), which image 301 compounds selected as likely to induce phenotypic effects in cells, and are performed in replicate by every source. Third, we curate a subset of 184 compounds from the Compound plates of cpg0016 (see Stratification for further details). Finally, every plate includes negative control wells treated with Dimethyl Sulfoxide (DMSO), the solvent used for all compound perturbations, which acts as a control when no compound is dissolved in DMSO. We curate negative controls from all plates we sampled POS-CTL, Target-2, and Compound wells from. Together, our dataset comprises 87,282 of the 1,096,069 wells in cpg0016.**"

- *In general, the manuscript would benefit from simplifying terminology and reducing redundancy. As mentioned before, dropping unnecessary terminology (e.g., causal representation learning, counterfactuals, etc) is important. And beyond that, using more compact descriptions and citing related work would improve the readability of the manuscript.*

We hope the above changes we have outlined in response to the reviewer's other comments – which include dropping terminology about casual representation learning, improving

discussion and citation about connections with prior works, and being more precise about data curation – both address the technical concerns and also improve readability of the manuscript. In addition, we have made overall concision changes to the manuscript.
