# Supplementary material for: Integrating chemical structures as treatments improves representations of microscopy images for morphological profiling
Source: PLoS Comput Biol. 2026 Jul 29;22(7):e1014483. doi: 10.1371/journal.pcbi.1014483 (PMC13446733; doi:10.1371/journal.pcbi.1014483)
Supplement: Supplementary file 5 [file pcbi.1014483.s011.pdf]

We once again thank the reviewers for their thoughtful feedback. In response to their points, we have incorporated a DINOv3 baseline without any fine-tuning, added an in-distribution test of MoA enrichment on the CP JUMP compound plates, and made further clarity and writing changes to our manuscript.

We hope with these updates, the manuscript is now suitable for publication in PLOS Computational Biology. Below, we respond to specific points from each reviewer (our responses in blue).

Reviewer #1: The revisions made have improved the manuscript, and my previous concerns have been globally addressed. In its current form, I support the acceptance of this work.

I would be interested to see the performance of DINOv3 used directly in a transfer learning setting to assess the need for fine-tuning.

We have incorporated this additional baseline. Figures 4 and 5 now include DINOv3 without any fine-tuning (reproduced below), which perform significantly worse than the fine-tuned DINOv3 model.

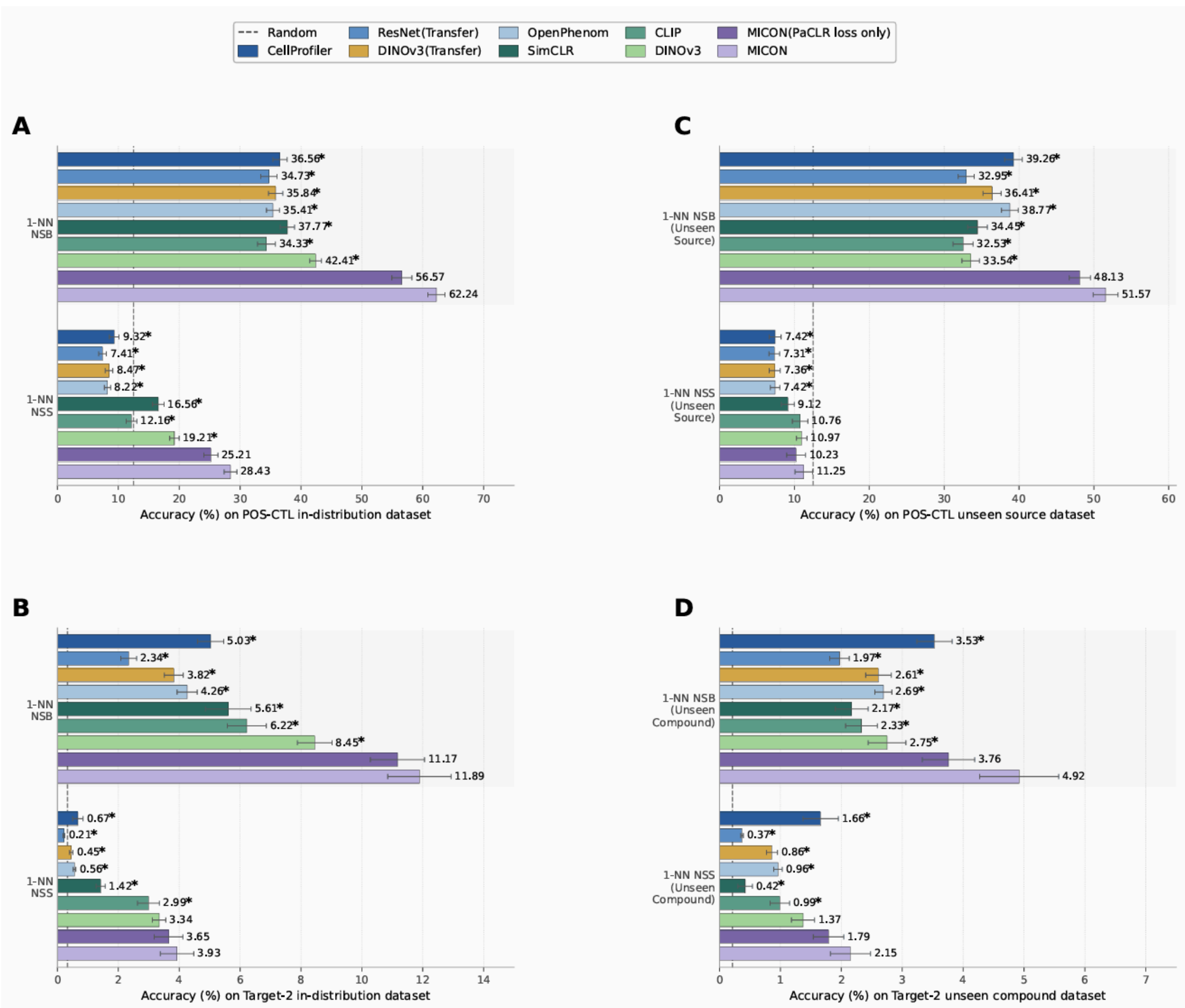

Fig 4. Compound-replicate matching accuracy for representation learning methods across evaluation settings. Retrieval accuracies on the (A) POS-CTL ID query dataset, (B) Target-2 ID query dataset, (C) POS-CTL OOD query dataset (unseen source), and (D) Target-2 OOD query dataset (unseen compounds). NSB designates retrieval of the nearest neighbor not in the same batch; NSS designates retrieval of the nearest neighbor not in the same source. n=3 models trained and evaluated with different dataset stratifications and different random seeds; the mean accuracy is labeled, and the error bars represent standard deviation. Asterisk indicates that MICON significantly outperforms the baseline, defined as $p < 0.05$ on an unpaired one-tailed t-test with performance across the n=3 random seeds as trials.

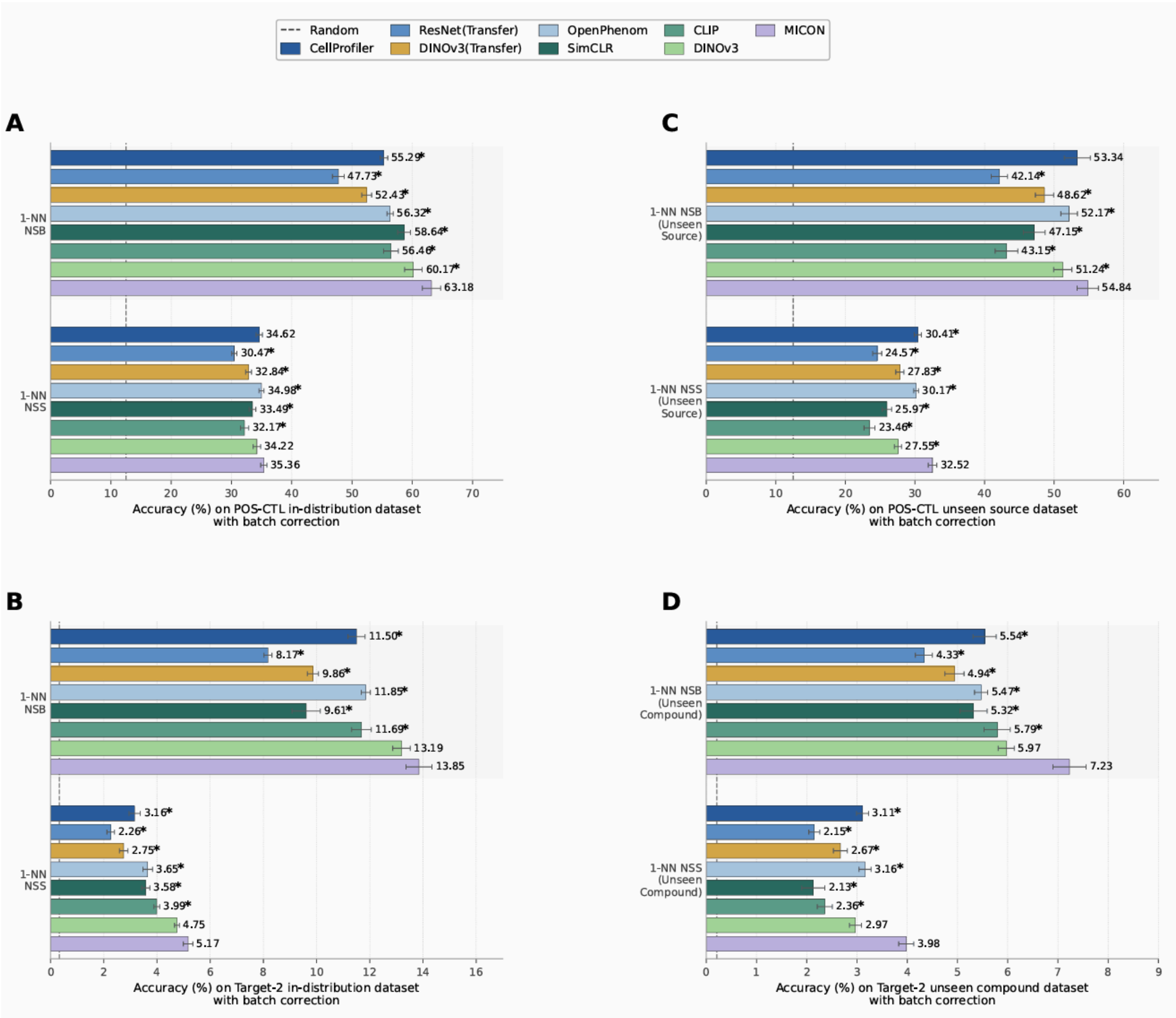

Fig 5. Compound-replicate matching accuracy for representation learning methods across evaluation settings, when microscopy batch effect correction is applied via spherizing. Retrieval accuracies on the (A) POS-CTL ID query dataset, (B) Target-2 ID query dataset, (C) POS-CTL OOD query dataset (unseen source), and (D) Target-2 OOD query dataset (unseen compounds). NSB designates retrieval of the nearest neighbor not in the same batch; NSS designates retrieval of the nearest neighbor not in the same source. n=3 models trained and evaluated with different dataset stratifications and different random seeds; the mean accuracy is labeled, and the error bars represent standard deviation.. Asterisk indicates that MICON significantly outperforms the baseline, defined as $p < 0.05$ on an unpaired one-tailed t-test with performance across the n=3 random seeds as trials.

Reviewer #2: The authors do an admirable job in their response, adding substantially to the benchmarking, adding an honest assessment of the counterfactual generation capabilities, and increasing clarity throughout the manuscript. Only a few comments below.

1. The new data in Table 1 raises a fundamental question about the method's premise. The authors hypothesize that 'integrating chemical structures... improves learned representations.' Yet, in Table 1, the image-only baseline (SimCLR) achieves a higher Average Fold-of-Enrichment than MICON. It is also very interesting that CellProfiler finds different enriched compounds. While other analyses do see improvements (sometimes marginal), the authors should consider tamping down claims in the abstract and author summary highlighting the benefits of the chemical encoder.

We thank the reviewers for this point. In conjunction with Reviewer #3's points, which raised the issue that we conducted the mechanism of action enrichment task on an out-of-distribution dataset, we reproduced this analysis using compound plates from CP JUMP. In a more in-distribution set-up, MICON has a higher mean odds ratio and enriches for more compounds (see the new Table 1 reproduced below). This suggests that MICON is successful in conferring benefits in identifying biological signal in more in-distribution datasets, but not necessarily out-of-distribution. This interpretation is consistent with what we observe in compound replicate retrieval: in our most challenging setting where sources are held-out, even though MICON outperforms other baselines, it still performs worse than random guessing. Together, this suggests that both aspects necessitated by representation learning - biological relevance and technical robustness - are challenging when operating out-of-distribution.

Table 1. Mechanism of action enrichment for the cpg0016 compound dataset. We report the average fold-of-enrichment with imputation (imputing the size of the background set as in [24]) and the percent of significant compounds.

	Average Fold-of-Enrichment	Percent Significant Compounds
CellProfiler	20.69	11.45%
SimCLR	21.07	14.39%
CLIP	27.47	14.60%
DINOv3	35.39	15.97%
MICON	37.52	16.18%

We have updated the manuscript to include these new experiments and incorporate the above interpretation:

"We evaluated MoA enrichment on a curated subset of the cpg0016 compound plates, using spherized features extracted by each of our trained models plus CellProfiler features (Table 1)...

We observe that MICON achieves a higher performance, both on average fold-of-enrichment and percent significant compounds than other methods, suggesting that the improvements in performance MICON achieved in compound-replicate retrieval

also translate into better clustering of compounds with similar mechanisms of action. All self-supervised models (SimCLR, CLIP, DINOv3, and MICON) outperformed CellProfiler features by a significant margin by both metrics, unlike our compound-replicate matching experiments. This may be because unlike compound-replicate retrieval, MoA enrichment metrics allow for retrieval of compounds from the same batch, making it less challenging of a batch effect setting."

We also revise the introduction and abstract to make it clearer that most performance for our method seems to stem from our representation learning framework, with the chemical compound encoder providing a small, but significant improvement on top of this:

"We demonstrate that incorporating chemical compound information into the learning process provides **small, but consistent improvements** in performance and that modeling compounds specifically as treatments outperforms approaches that directly align images and compounds in a single representation space."

"Second, through ablation of MICON's multimodal component, we demonstrate that integrating chemical compound information provides a **small but statistically significant** improvement in performance relative to learning from images alone."

2. In light of this result, the authors should discuss the limitations of MICON more prominently. How much extra compute is required? What if the perturbation is not chemical? Are the representations biologically interpretable?

Because of the lightweight nature of the chemical compound encoder, the computational cost is negligible. However, we agree there are limitations from a generality perspective, and interesting opportunities to analyze the chemical encoder representations that we have not followed up on in this work. We have updated the discussion with these points:

"**Our current proposed model is restricted to chemical compounds as input perturbations.** Extending MICON to additional perturbation modalities, such as genetic or environmental perturbations, could similarly model them as treatments that induce a transformation of images."

"**Finally, there are opportunities in interpretation that we have not explored in this work: future work should investigate if the compound encoder attends to parts of chemical compounds that are responsible for biological mechanism of action.**"

3. The authors improved clarity, but we recommend some additional tweaks. For example, Figure 1 is clearer, but the "predicted PACLR loss" label is still slightly confusing. This labeling confusion persists in the result section when the authors say "ablated the predicted PaCLR loss". The loss is not actually predicted (as the text

describes) but instead we believe the authors to mean the “PACLR loss on predicted images”. Additionally, the legend in Sup Fig 1 is a bit confusing. When the authors write “averaged CellProfiler features” do they mean “aggregated well-level profiles of CellProfiler features”. Are these profiles post feature selection?

We appreciate the reviewer pointing out these areas where the paper lacks clarity - we have made these modifications throughout the manuscript.

4. We were able to successfully access the huggingface resources, we thank the authors for including this important detail.

Reviewer #3: The new version of the paper is more clear and readable. The authors have incorporated the suggested revisions.

The only remaining issue in the new version is that the evaluation of biological signal using mechanism of action prediction as a task, does not show any improvements or benefits with the proposed approach. This contradicts the title of the paper, which claims that the representations are improved for morphological profiling. Replicate matching is only an experimental quality control metric, but it does not solve any useful biological query. Therefore, if the proposed method only improves replicate matching and not downstream biological tasks, the significance of the method is diminished. As it is now, the results indicate that the proposed approach is a preliminary idea that needs more investigation because it only improves the technical quality of imaging features and not the biological relevance.

Questions:

* Why was the MOA task evaluated on a different dataset? There are subsets of the JUMP-CP dataset that have biological annotations which have been used previously for self-supervised research (see e.g., ref 9).
* Previous work has investigated the effects of batch correction in both the technical aspects and the biological aspects. This study seems incomplete by focusing primarily on replicate matching and leaving biological relevance on the sides. Previously proposed benchmarks such as the cited work of Arevalo et al. (2024) have many combinations of scenarios that could be used as benchmarking tests for MICON. Can the authors evaluate their proposed methods on these tasks?
* If MICON only works on the subset selected by the authors, the impact of the proposed methodology is limited. Can the authors investigate their approach following evaluation benchmarks proposed for the JUMP-CP dataset, which is the main dataset used in this work?

We thank the reviewer for suggesting further benchmarks. We have now evaluated the mechanism of action enrichment task using a subset of the compound plates from JUMP-CP. In a more in-distribution set-up, MICON has a higher mean odds ratio and

significantly enriches for more compounds (see the new Table 1 reproduced below). This suggests that MICON is successful in conferring benefits in identifying biological signal in more in-distribution datasets, but not necessarily out-of-distribution. This interpretation is consistent with what we observe in compound replicate retrieval: in our most challenging setting where sources are held-out, even though MICON outperforms other baselines, it still performs worse than random guessing. Together, this suggests that both aspects necessitated by representation learning - biological relevance and technical robustness - are challenging when operating out-of-distribution.

We have updated the manuscript to include these new experiments and incorporate the above interpretation:

Table 1. Mechanism of action enrichment for the cpg0016 compound dataset. We report the average fold-of-enrichment with imputation (imputing the size of the background set as in [24]) and the percent of significant compounds.

	Average Fold-of-Enrichment	Percent Significant Compounds
CellProfiler	20.69	11.45%
SimCLR	21.07	14.39%
CLIP	27.47	14.60%
DINOv3	35.39	15.97%
MICON	37.52	16.18%

We have updated the manuscript to include these new experiments and incorporate the above interpretation:

“We evaluated MoA enrichment on a curated subset of the cpg0016 compound plates, using spherized features extracted by each of our trained models plus CellProfiler features (Table 1)...

We observe that MICON achieves a higher performance, both on average fold-of-enrichment and percent significant compounds than other methods, suggesting that the improvements in performance MICON achieved in compound-replicate retrieval also translate into better clustering of compounds with similar mechanisms of action. All self-supervised models (SimCLR, CLIP, DINOv3, and MICON) outperformed CellProfiler features by a significant margin by both metrics, unlike our compound-replicate matching experiments. This may be because unlike compound-replicate retrieval, MoA enrichment metrics allow for retrieval of compounds from the same batch, making it less challenging of a batch effect setting.”

Regarding the evaluations of Arevalo *et al*., we believe our mechanism of action enrichment tasks are a better way to demonstrate sensitivity to biological signal. First, Arevalo *et al*. assess clustering based upon compound identity. This means that what they report as biometrics is using the same signal we exploit for compound-replicate retrieval. Second, we believe that the nearest-neighbor metrics we report are more robust than the clustering metrics reported by Arevalo *et al*. In their work, they adapt single cell transcriptomics clustering metrics, which were originally proposed on

datasets with tens of classes with thousands of samples per class, to datasets with 80,000+ classes and a mode of 2 samples per class. This renders some of the proposed clustering metrics ill-posed. We have forthcoming work (Hayir *et al*. 2026, in preparation) that demonstrates that for these settings, across some metrics, performance differences between methods benchmarked by Arevalo *et al*. can be entirely attributed to differences in latent dimensionality between methods (specifically, we randomly shuffle labels, and show differences in metrics remain unchanged). As a full exploration of these challenges is outside of the scope of this work, we prefer to exclude the benchmark entirely from this work.
