# Supplementary material for: Integrating chemical structures as treatments improves representations of microscopy images for morphological profiling
Source: PLoS Comput Biol. 2026 Jul 29;22(7):e1014483. doi: 10.1371/journal.pcbi.1014483 (PMC13446733; doi:10.1371/journal.pcbi.1014483)
Supplement: Supplementary file 6 [file pcbi.1014483.s009.docx]

Yu et al. present MICON, a novel self-supervised learning framework that enhances image-based profiling by integrating chemical compound structure into microscopy image representations through a causal counterfactual approach. The authors demonstrate that MICON outperforms both traditional hand-crafted features from CellProfiler and various deep learning baselines across challenging evaluation settings, including out-of-distribution generalization to unseen compounds and imaging sources using publicly available data from the JUMP-CP consortium. Modeling chemical treatments as causal interventions using a fusion model is a key conceptual advance that distinguishes this work. The work is written elegantly, and the methods and analyses sound. We describe these comments in more details below, but the evaluation relies on retrieval accuracy, and additional assessment of biological relevance (e.g., phenotypic similarity analyses or performance on downstream tasks like mechanism-of-action prediction) would further strengthen the study’s impact for the PLOS Computational Biology audience. Moreover, benchmarking against recent multimodal or batch correction methods, including CLOOME and MolPhenix, as well as other hand-crafted feature approaches such as SPACe, would provide a more compelling and comprehensive comparison. Given the low performance of the approach, which we do not consider a major downside for publication, it would be particularly interesting to add these other techniques, which we suspect would produce similarly low performance. Overall, this work represents a strong contribution to image-based profiling and multimodal representation learning, with minor revisions recommended to align fully with the journal’s scope and expectations and increase reach of the benchmarking framework described herein.

Comments. We do not believe these are critical to accepting the manuscript, but highly recommend making these additions. The manuscript is already methodologically sound.

1. Regarding evaluation of counterfactual representations. While MICON introduces a novel approach for generating counterfactual representations, its evaluation currently centers on nearest-neighbor retrieval accuracy. The authors should consider adding a more direct assessment of biological meaning. This assessment could involve comparing the phenotypic similarity distributions between generated counterfactuals and real perturbation images. Would it be possible to show a generated image? Visualization and quantitative comparison methods, such as UMAP projections, phenotypic clustering, or statistical/image reconstruction measures like Kolmogorov–Smirnov (KS) statistics, Earth Mover’s Distance, PSNR, and LPIPs could further help determine how well these representations preserve biologically meaningful variation in relation to the other methods. Further, incorporating higher-level biological annotations (such as compound mechanisms of action (MoA) or known phenotypic groupings) would more clearly validate the relevance of the generated representations. Such analyses would not only demonstrate the realism of the counterfactuals but also their interpretability in a biological context.
2. Benchmarking other approaches. The manuscript would benefit from a broader comparative evaluation against recent state-of-the-art deep learning frameworks. Notably, adding SPACe as an additional baseline for hand-crafted features would be particularly informative, given its strong performance on JUMP-CP datasets, and outperformance of CellProfiler features. Additionally, multimodal methods such as CLOOME and MolPhenix integrate compound structure with image-based data in ways that are conceptually related to MICON’s approach. While direct comparisons may be complicated by differences in architecture or pretraining strategies, a more detailed discussion, or even a proxy evaluation using similar training objectives, would clarify the distinct advantages of MICON’s causal integration strategy. Such analysis would strengthen the empirical foundation of the work and better contextualize its contributions within the evolving landscape of multimodal representation learning. Refocusing the methodologies presented here as a benchmark and adding these methods would add a lot of oomph.

Minor comments

1. Additional detail is needed regarding the final loss formulation. Specifically, it is unclear whether the PAC and PaCLR loss are equally weighted in the overall objective. Were alternative weighting schemes considered or tuned during development? Would there be any benefit to parameterizing the contribution of each component? Clarifying this aspect would help readers better understand the relative contribution of each component to the optimization process and how the model balances learning from real versus counterfactual examples.
2. There were a few typos. Figure 1 legend: “pertubation” should be corrected to “perturbation.” Line 54: “ifmages” is a typo; should be “images.” Line 90: “pertubation-aware” should be corrected to “perturbation-aware.” Supplementary Figure 3: “magneta” should be corrected to “magenta.”
3. Figure 1 is very clear and effective, but we wonder why the matrix is duplicated in panel C, does this have meaning?
4. Reference to a specific section missing in line 106, also lines 153, 235, 282, 388.
5. It would be helpful to justify choice of cosine distance in simCLR loss function on line 128
6. It would be helpful to clarify if the method treats FOVs from the same image in the same way as FOVs from different images (Line 132-134)
7. The authors present a wonderfully detailed and accurate description of JUMP.
8. The data splitting procedures and evaluation strategies for them are elegantly designed. However, I’m wondering if the 184 compounds unseen during training could be split even further based on target or mechanism of action. For example, It would be more impressive if the model would work on an entirely new MOA.
9. We did not anticipate seeing accuracy so low! It would be helpful to discuss this sooner, in the abstract (or even in the title). It would also be helpful to discuss in what cases this approach would be useful. Similarly, maybe reporting top3 or top5 matching would be helpful?
10. It is refreshing to see Figure 6 results and interpretation regarding the comparison to CellProfiler after batch effect correction seeing minimal performance improvements. Kudos!
11. The authors include a dropbox link for model weights and representations. This is brittle and liable to 404 at any time. We recommend that the authors instead deposit this data in a third-party resource (e.g., figshare), which will perpetuate and generate a DOI.

This is a signed review.

Erik Serrano

Gregory P. Way, PhD

University of Colorado Department of Biomedical Informatics
