# Supplementary material for: Integrating chemical structures as treatments improves representations of microscopy images for morphological profiling
Source: PLoS Comput Biol. 2026 Jul 29;22(7):e1014483. doi: 10.1371/journal.pcbi.1014483 (PMC13446733; doi:10.1371/journal.pcbi.1014483)
Supplement: Peer review history

# Peer review: Decision Letter 0

**DOI:** 10.1371/journal.pcbi.1014483.r001

**Authors:** Virginia E. Pitzer, Virginie Uhlmann

7 Jul 2025

PCOMPBIOL-D-25-00920

Causal integration of chemical structures improves representations of microscopy images for morphological profiling

PLOS Computational Biology

Dear Dr. Lu,

Thank you for submitting your manuscript to PLOS Computational Biology. After careful consideration, we feel that it has merit but does not fully meet PLOS Computational Biology's publication criteria as it currently stands. Therefore, we invite you to submit a revised version of the manuscript that addresses the points raised during the review process.

Please submit your revised manuscript within 60 days Sep 06 2025 11:59PM. If you will need more time than this to complete your revisions, please reply to this message or contact the journal office at ploscompbiol@plos.org. When you're ready to submit your revision, log on to https://www.editorialmanager.com/pcompbiol/ and select the 'Submissions Needing Revision' folder to locate your manuscript file.

Please include the following items when submitting your revised manuscript:

* A rebuttal letter that responds to each point raised by the editor and reviewer(s). You should upload this letter as a separate file labeled 'Response to Reviewers'. This file does not need to include responses to formatting updates and technical items listed in the 'Journal Requirements' section below.

* A marked-up copy of your manuscript that highlights changes made to the original version. You should upload this as a separate file labeled 'Revised Manuscript with Track Changes'.

* An unmarked version of your revised paper without tracked changes. You should upload this as a separate file labeled 'Manuscript'.

If you would like to make changes to your financial disclosure, competing interests statement, or data availability statement, please make these updates within the submission form at the time of resubmission. Guidelines for resubmitting your figure files are available below the reviewer comments at the end of this letter

We look forward to receiving your revised manuscript.

Kind regards,

Virginie Uhlmann

Academic Editor

PLOS Computational Biology

Virginia Pitzer

Editor-in-Chief

PLOS Computational Biology

**Additional Editor Comments (if provided):**

**Journal Requirements:**

[Note: HTML markup is below. Please do not edit.]

**Reviewers' comments:**

Reviewer's Responses to Questions

**Comments to the Authors:**

**Please note here if the review is uploaded as an attachment.**

Reviewer #1: The review is uploaded as an attachment.

Reviewer #2: Review attached

Reviewer #3: The manuscript presents a machine learning strategy to obtain representations of cellular images that are robust to batch effects. The proposed strategy is based on contrastive learning, and makes extensive use of knowledge about experimental design to select positive and negative pairs for training. In addition, the strategy proposes to generate synthetic representations that combine chemical structures and image features that simulate treatment effects in the latent space. The experimental results indicate that the proposed strategy is more robust to batch effects and has the potential to provide improved quantitative measures for image-based profiling.

While the manuscript is interesting and has promising results, it requires major revisions in presentation and clarifications to put it in context with respect to existing literature (see comments below).

Comments

1. Causal integration

* The authors claim that their framework is a causal representation learning framework for morphological profiling (lines 60-62). This claim is not accurate in the context of recent machine learning work that investigates causal representation learning from the perspective of causal inference, causal structural models, and Pearl’s ladder of causation (see e.g., [A]). Given that the proposed method is not aligned with these efforts, it is recommended to drop such terminology from the paper.

* The proposed methodology is clearly inspired by prior knowledge about the experimental design of perturbation experiments, serving as an effective prior to improve performance. However, the current terminology around causal representation learning and counterfactuals is confusing, and does not accurately reflect what the methodology is about. In practice, the proposed strategy is a sampling strategy that carefully selects minibatches with positive and negative examples that make sense for the problem. The method does not incorporate explicit inductive biases in the architectures or probabilistic mechanisms of the model, nor does it implement any type of causal inference in practice. Making the link with causal representation learning and counterfactuals is a stretch of terminology that does not benefit either the manuscript or the community.

* The use of experimental metadata has been extensively explored in previous literature too. For instance, [B] and [C] extend self-supervised methods to leverage images of cells with the same treatment. These ideas are also connected with previous literature that use weakly supervised learning for representation learning [D]. It is recommended that the authors acknowledge these connections and explain the differences, as the current manuscript seems to claim that using such metadata is a novel idea.

* The causal model in Figure 2 is similar to, but not consistent with previous literature in the field. For instance, [D] and [E] present and formalize an identical graph, which differs from the variables and causal relationships presented in this manuscript. Why is that difference? This manuscript does not justify the choice, does not explain the differences, and ignores these previous efforts. This suggests that the adoption of causal terminology is rather rushed and not considered carefully in the context of existing literature. If a causal graph is needed, it is recommended that the authors adopt the previously existing model or formalize a new one based on evolving needs for the proposed model (which does not seem to be the case).

* Regarding counterfactual representations, the model is not really performing counterfactual inference to deduce these representations. The model design is more consistent with a conditional generative model, with chemical structures and image features as a prompt, and new feature representations as a response. This is similar to the design of contemporary generative models, such as language models, where given a prompt, consistent outputs are generated, without the responses being necessarily counterfactuals. In fact, it is believed that the outputs are the result of interpolations of knowledge possessed by the models instead of counterfactual reasoning. In the proposed approach, batches and other variables of the causal model are not explicitly considered in the process. Counterfactual computation is a specific branch of active research in statistics and machine learning and the proposed model is not consistent with such literature. It is recommended to drop the counterfactual terminology and present it as an estimation of treated representations instead.

2. Experimental evaluation

* The experimental results presented in the paper are extremely interesting, as they demonstrate that careful training of models can make a difference in robustness to batch effects. The manuscript does an excellent job at testing out of distribution performance by leaving batches and sources out.

* It is recommended to report what is the performance of ResNet features only without the proposed model. This can establish a baseline to estimate the contribution of the components of the model. Additional similar ablations are also recommended.

* The central assumption of the manuscript is that same-compound retrieval is a good proxy for representation learning evaluation. However, this type of evaluation only checks replicability of the image signal, but not its biological significance. In other words, compounds that have similar effects may be represented with perfect clusters of replicates, but may be far apart in the feature space even when they are supposed to be related. This type of biological outcome evaluation is typically implemented with mechanism of action prediction tasks, for instance. It is recommended to implement such metrics in the analysis to confirm that the model is not just optimizing clusters of replicates, but rather finding meaningful connections between treatments.

* To back-up the claims that the synthetic representations can simulate treatment effects, it is important to evaluate biologically relevant tasks, such as mechanism of action prediction (see previous point). It is unclear whether the model can actually simulate treatments or just mimic representations that are expected in a cluster of replicates. Qualitative evaluations can be helpful in addition to the metrics, to illustrate that compounds with similar MoAs actually share similar representations.

* The evaluation with biologically relevant labels (e.g., mechanism of action) may require additional data beyond the strong treatments selected for this study. This is also a potential limitation of the manuscript that can be addressed by looking at increasingly more complex queries and references.

* The manuscript claims that their evaluation is the most rigorous. However, previous work [F] has proposed more extensive evaluation frameworks on the same dataset, considering generalization scenarios and biological tasks that are left out of this manuscript. Please, consider adopting those metrics, or adjusting the language to acknowledge these other efforts.

3. Writing and presentation.

* The description of the dataset presents details of the full JUMP dataset, which is not really used in the manuscript. This is unnecessary and misleading. The JUMP dataset can be simply cited and the subsets described, without inflating expectations about what the model was tested on.

* In general, the manuscript would benefit from simplifying terminology and reducing redundancy. As mentioned before, dropping unnecessary terminology (e.g., causal representation learning, counterfactuals, etc) is important. And beyond that, using more compact descriptions and citing related work would improve the readability of the manuscript.

[A] https://arxiv.org/abs/2102.11107

[B] https://openaccess.thecvf.com/content/CVPR2024W/CVMI/papers/Yao_Weakly_Supervised_Set-Consistency_Learning_Improves_Morphological_Profiling_of_Single-Cell_Images_CVPRW_2024_paper.pdf

[C] https://arxiv.org/pdf/2209.07819

[D] https://www.nature.com/articles/s41467-024-45999-1

[E] https://www.biorxiv.org/content/10.1101/2024.12.23.630105v1

[F] https://www.nature.com/articles/s41467-024-50613-5

**********

**Have the authors made all data and (if applicable) computational code underlying the findings in their manuscript fully available?**

The PLOS Data policy requires authors to make all data and code underlying the findings described in their manuscript fully available without restriction, with rare exception (please refer to the Data Availability Statement in the manuscript PDF file). The data and code should be provided as part of the manuscript or its supporting information, or deposited to a public repository. For example, in addition to summary statistics, the data points behind means, medians and variance measures should be available. If there are restrictions on publicly sharing data or code —e.g. participant privacy or use of data from a third party—those must be specified.

Reviewer #1: Yes

Reviewer #2: Yes

Reviewer #3: Yes

**********

PLOS authors have the option to publish the peer review history of their article (what does this mean?). If published, this will include your full peer review and any attached files.

If you choose “no”, your identity will remain anonymous but your review may still be made public.

**Do you want your identity to be public for this peer review?**  For information about this choice, including consent withdrawal, please see our Privacy Policy.

Reviewer #1: No

Reviewer #2: **Yes:**  Erik Serrano and Gregory P. Way, PhD

Reviewer #3: No

[NOTE: If reviewer comments were submitted as an attachment file, they will be attached to this email and accessible via the submission site. Please log into your account, locate the manuscript record, and check for the action link "View Attachments". If this link does not appear, there are no attachment files.]

**Figure resubmission:**

While revising your submission, please upload your figure files to the Preflight Analysis and Conversion Engine (PACE) digital diagnostic tool, https://pacev2.apexcovantage.com/. PACE helps ensure that figures meet PLOS requirements. To use PACE, you must first register as a user. Registration is free. Then, login and navigate to the UPLOAD tab, where you will find detailed instructions on how to use the tool. If you encounter any issues or have any questions when using PACE, please email PLOS at figures@plos.org. Please note that Supporting Information files do not need this step. If there are other versions of figure files still present in your submission file inventory at resubmission, please replace them with the PACE-processed versions.

**Reproducibility:**

To enhance the reproducibility of your results, we recommend that authors of applicable studies deposit laboratory protocols in protocols.io, where a protocol can be assigned its own identifier (DOI) such that it can be cited independently in the future. Additionally, PLOS ONE offers an option to publish peer-reviewed clinical study protocols. Read more information on sharing protocols at https://plos.org/protocols?utm_medium=editorial-email&utm_source=authorletters&utm_campaign=protocols

Attachment Submitted filename: 2025 MICON review.pdf

Attachment Submitted filename: yu_etal_compiled_review.docx

# Author comment: Author response to Decision Letter 1

**DOI:** 10.1371/journal.pcbi.1014483.r002

22 Dec 2025

Attachment Submitted filename: micon_response_letter.pdf

# Peer review: Decision Letter 1

**DOI:** 10.1371/journal.pcbi.1014483.r003

**Authors:** Virginia E. Pitzer, Virginie Uhlmann

2 Feb 2026

PCOMPBIOL-D-25-00920R1

Integrating chemical structures as treatments improves representations of microscopy images for morphological profiling

PLOS Computational Biology

Dear Dr. Lu,

Thank you for submitting your manuscript to PLOS Computational Biology. After careful consideration, we feel that it has merit but does not fully meet PLOS Computational Biology's publication criteria as it currently stands. Therefore, we invite you to submit a revised version of the manuscript that addresses the points raised during the review process.

Please submit your revised manuscript by Apr 04 2026 11:59PM. If you will need more time than this to complete your revisions, please reply to this message or contact the journal office at ploscompbiol@plos.org. When you're ready to submit your revision, log on to https://www.editorialmanager.com/pcompbiol/ and select the 'Submissions Needing Revision' folder to locate your manuscript file.

Please include the following items when submitting your revised manuscript:

* A letter that responds to each point raised by the editor and reviewer(s). You should upload this letter as a separate file labeled 'Response to Reviewers'. This file does not need to include responses to formatting updates and technical items listed in the 'Journal Requirements' section below.

* A marked-up copy of your manuscript that highlights changes made to the original version. You should upload this as a separate file labeled 'Revised Manuscript with Track Changes'.

* An unmarked version of your revised paper without tracked changes. You should upload this as a separate file labeled 'Manuscript'.

If you would like to make changes to your financial disclosure, competing interests statement, or data availability statement, please make these updates within the submission form at the time of resubmission. Guidelines for resubmitting your figure files are available below the reviewer comments at the end of this letter.

We look forward to receiving your revised manuscript.

Kind regards,

Virginie Uhlmann

Academic Editor

PLOS Computational Biology

Virginia Pitzer

Editor-in-Chief

PLOS Computational Biology

**Additional Editor Comments (if provided):**

Please consider modifying the title of the article to address the major concern from Reviewer 3.

**Journal Requirements:**

If the reviewer comments include a recommendation to cite specific previously published works, please review and evaluate these publications to determine whether they are relevant and should be cited. There is no requirement to cite these works unless the editor has indicated otherwise.

[Note: HTML markup is below. Please do not edit.]

**Reviewers' comments:**

Reviewer's Responses to Questions

**Comments to the Authors:**

**Please note here if the review is uploaded as an attachment.**

Reviewer #1: The revisions made have improved the manuscript, and my previous concerns have been globally addressed. In its current form, I support the acceptance of this work.

I would be interested to see the performance of DINOv3 used directly in a transfer learning setting to assess the need for fine-tuning.

Finally, I would like to note that I particularly appreciated the core idea of the proposed method.

Reviewer #2: The authors do an admirable job in their response, adding substantially to the benchmarking, adding an honest assessment of the counterfactual generation capabilities, and increasing clarity throughout the manuscript. Only a few comments below.

1. The new data in Table 1 raises a fundamental question about the method's premise. The authors hypothesize that 'integrating chemical structures... improves learned representations.' Yet, in Table 1, the image-only baseline (SimCLR) achieves a higher Average Fold-of-Enrichment than MICON. It is also very interesting that CellProfiler finds different enriched compounds. While other analyses do see improvements (sometimes marginal), the authors should consider tamping down claims in the abstract and author summary highlighting the benefits of the chemical encoder.

2. In light of this result, the authors should discuss the limitations of MICON more prominently. How much extra compute is required? What if the perturbation is not chemical? Are the representations biologically interpretable?

3. The authors improved clarity, but we recommend some additional tweaks. For example, Figure 1 is clearer, but the “predicted PACLR loss” label is still slightly confusing. This labeling confusion persists in the result section when the authors say “ablated the predicted PaCLR loss”. The loss is not actually predicted (as the text describes) but instead we believe the authors to mean the “PACLR loss on predicted images”. Additionally, the legend in Sup Fig 1 is a bit confusing. When the authors write “averaged CellProfiler features” do they mean “aggregated well-level profiles of CellProfiler features”. Are these profiles post feature selection?

4. We were able to successfully access the huggingface resources, we thank the authors for including this important detail.

Reviewer #3: The new version of the paper is more clear and readable. The authors have incorporated the suggested revisions.

The only remaining issue in the new version is that the evaluation of biological signal using mechanism of action prediction as a task, does not show any improvements or benefits with the proposed approach. This contradicts the title of the paper, which claims that the representations are improved for morphological profiling. Replicate matching is only an experimental quality control metric, but it does not solve any useful biological query. Therefore, if the proposed method only improves replicate matching and not downstream biological tasks, the significance of the method is diminished. As it is now, the results indicate that the proposed approach is a preliminary idea that needs more investigation because it only improves the technical quality of imaging features and not the biological relevance.

Questions:

* Why was the MOA task evaluated on a different dataset? There are subsets of the JUMP-CP dataset that have biological annotations which have been used previously for self-supervised research (see e.g., ref 9).

* Previous work has investigated the effects of batch correction in both the technical aspects and the biological aspects. This study seems incomplete by focusing primarily on replicate matching and leaving biological relevance on the sides. Previously proposed benchmarks such as the cited work of Arevalo et al. (2024) have many combinations of scenarios that could be used as benchmarking tests for MICON. Can the authors evaluate their proposed methods on these tasks?

* If MICON only works on the subset selected by the authors, the impact of the proposed methodology is limited. Can the authors investigate their approach following evaluation benchmarks proposed for the JUMP-CP dataset, which is the main dataset used in this work?

**********

**Have the authors made all data and (if applicable) computational code underlying the findings in their manuscript fully available?**

The PLOS Data policy requires authors to make all data and code underlying the findings described in their manuscript fully available without restriction, with rare exception (please refer to the Data Availability Statement in the manuscript PDF file). The data and code should be provided as part of the manuscript or its supporting information, or deposited to a public repository. For example, in addition to summary statistics, the data points behind means, medians and variance measures should be available. If there are restrictions on publicly sharing data or code —e.g. participant privacy or use of data from a third party—those must be specified.

Reviewer #1: Yes

Reviewer #2: Yes

Reviewer #3: Yes

**********

PLOS authors have the option to publish the peer review history of their article (what does this mean?). If published, this will include your full peer review and any attached files.

If you choose “no”, your identity will remain anonymous but your review may still be made public.

**Do you want your identity to be public for this peer review?**  For information about this choice, including consent withdrawal, please see our Privacy Policy.

Reviewer #1: No

Reviewer #2: No

Reviewer #3: No

[NOTE: If reviewer comments were submitted as an attachment file, they will be attached to this email and accessible via the submission site. Please log into your account, locate the manuscript record, and check for the action link "View Attachments". If this link does not appear, there are no attachment files.]

**Figure resubmission:**

-->While revising your submission, we strongly recommend that you use PLOS’s NAAS tool (https://ngplosjournals.pagemajik.ai/artanalysis) to test your figure files. NAAS can convert your figure files to the TIFF file type and meet basic requirements (such as print size, resolution), or provide you with a report on issues that do not meet our requirements and that NAAS cannot fix.

After uploading your figures to PLOS’s NAAS tool - https://ngplosjournals.pagemajik.ai/artanalysis, NAAS will process the files provided and display the results in the "Uploaded Files" section of the page as the processing is complete. If the uploaded figures meet our requirements (or NAAS is able to fix the files to meet our requirements), the figure will be marked as "fixed" above. If NAAS is unable to fix the files, a red "failed" label will appear above. When NAAS has confirmed that the figure files meet our requirements, please download the file via the download option, and include these NAAS processed figure files when submitting your revised manuscript.

**Reproducibility:**

To enhance the reproducibility of your results, we recommend that authors of applicable studies deposit laboratory protocols in protocols.io, where a protocol can be assigned its own identifier (DOI) such that it can be cited independently in the future. Additionally, PLOS ONE offers an option to publish peer-reviewed clinical study protocols. Read more information on sharing protocols at https://plos.org/protocols?utm_medium=editorial-email&utm_source=authorletters&utm_campaign=protocols

# Author comment: Author response to Decision Letter 2

**DOI:** 10.1371/journal.pcbi.1014483.r004

18 May 2026

Attachment Submitted filename: MICON-rev2-reviewer-response-letter.pdf

# Peer review: Decision Letter 2

**DOI:** 10.1371/journal.pcbi.1014483.r005

**Authors:** Virginia E. Pitzer, Virginie Uhlmann

22 Jun 2026

Dear Dr. Lu,

We are pleased to inform you that your manuscript 'Integrating chemical structures as treatments improves representations of microscopy images for morphological profiling' has been provisionally accepted for publication in PLOS Computational Biology.

Before your manuscript can be formally accepted you will need to complete some formatting changes, which you will receive in a follow up email. A member of our team will be in touch with a set of requests.

Please note that your manuscript will not be scheduled for publication until you have made the required changes, so a swift response is appreciated.

IMPORTANT: The editorial review process is now complete. PLOS will only permit corrections to spelling, formatting or significant scientific errors from this point onwards. Requests for major changes, or any which affect the scientific understanding of your work, will cause delays to the publication date of your manuscript.

Should you, your institution's press office or the journal office choose to press release your paper, you will automatically be opted out of early publication. We ask that you notify us now if you or your institution is planning to press release the article. All press must be co-ordinated with PLOS.

Thank you again for supporting Open Access publishing; we are looking forward to publishing your work in PLOS Computational Biology.

Best regards,

Virginie Uhlmann

Academic Editor

PLOS Computational Biology

Virginia Pitzer

Editor-in-Chief

PLOS Computational Biology

***********************************************************

Reviewer's Responses to Questions

**Comments to the Authors:**

**Please note here if the review is uploaded as an attachment.**

Reviewer #3: It is disappointing that the authors chose to not report evaluation on existing benchmarks. Having these metrics reported would increase the impact and transparency of this research, and would be informative to assess progress in the community.

That being said, the paper is good enough for publication and no further changes are needed.

**********

**Have the authors made all data and (if applicable) computational code underlying the findings in their manuscript fully available?**

The PLOS Data policy requires authors to make all data and code underlying the findings described in their manuscript fully available without restriction, with rare exception (please refer to the Data Availability Statement in the manuscript PDF file). The data and code should be provided as part of the manuscript or its supporting information, or deposited to a public repository. For example, in addition to summary statistics, the data points behind means, medians and variance measures should be available. If there are restrictions on publicly sharing data or code —e.g. participant privacy or use of data from a third party—those must be specified.

Reviewer #3: Yes

**********

PLOS authors have the option to publish the peer review history of their article (what does this mean?). If published, this will include your full peer review and any attached files.

If you choose “no”, your identity will remain anonymous but your review may still be made public.

**Do you want your identity to be public for this peer review?**  For information about this choice, including consent withdrawal, please see our Privacy Policy.

Reviewer #3: No

# Editor report: Acceptance letter

**DOI:** 10.1371/journal.pcbi.1014483.r006

**Authors:** Virginia E. Pitzer, Virginie Uhlmann

PCOMPBIOL-D-25-00920R2

Integrating chemical structures as treatments improves representations of microscopy images for morphological profiling

Dear Dr Lu,

I am pleased to inform you that your manuscript has been formally accepted for publication in PLOS Computational Biology. Your manuscript is now with our production department and you will be notified of the publication date in due course.

The corresponding author will soon be receiving a typeset proof for review, to ensure errors have not been introduced during production. Please review the PDF proof of your manuscript carefully, as this is the last chance to correct any errors. Please note that major changes, or those which affect the scientific understanding of the work, will likely cause delays to the publication date of your manuscript.

Soon after your final files are uploaded, unless you have opted out, the early version of your manuscript will be published online. The date of the early version will be your article's publication date. The final article will be published to the same URL, and all versions of the paper will be accessible to readers.

For Research, Software, and Methods articles, you will receive an invoice from PLOS for your publication fee after your manuscript has reached the completed accept phase. If you receive an email requesting payment before acceptance or for any other service, this may be a phishing scheme. Learn how to identify phishing emails and protect your accounts at https://explore.plos.org/phishing.

Thank you again for supporting PLOS Computational Biology and open-access publishing. We are looking forward to publishing your work!

With kind regards,

Janani Seenivasan

PLOS Computational Biology | Carlyle House, Carlyle Road, Cambridge CB4 3DN | United Kingdom ploscompbiol@plos.org | Phone +44 (0) 1223-442824 | ploscompbiol.org | @PLOSCompBiol
